# Priority questions for the next decade of blue carbon science

**DOI:** 10.1038/s41559-026-03020-6

**Published:** 2026-03-24

**Authors:** Peter I. Macreadie, George E. Biddulph, Pere Masque, Hilary Kennedy, Jimena Samper-Villarreal, J. Patrick Megonigal, Hannah K. Morrissette, Tania E. Romero-Gonzalez, Vanessa Hatje, Jana Friedrich, Sigit D. Sasmito, Kenta Watanabe, Inés Mazarrasa, Dorte Krause-Jensen, Janine B. Adams, Miguel Cifuentes-Jara, Ariane Arias-Ortiz, Andre S. Rovai, Milica Stankovic, Kirsten Isensee, Ana M. Queirós, Luzhen Chen, Jorge Herrera-Silveira, Catriona L. Hurd, Rashid Ismail, Ken W. Krauss, Anna Lafratta, Maria M. Palacios, William E. N. Austin

**Affiliations:** 1 Centre for Nature Positive Solutions, Biology Department, School of Science, RMIT University, Melbourne, Victoria Australia; 2 Deakin Marine Research and Innovation Centre, School of Life and Environmental Sciences, Deakin University, Burwood, Victoria Australia; 3 School of Geography and Sustainable Development, University of St. Andrews, St. Andrews, UK; 4 School of Science and Centre for Marine Ecosystems Research, Edith Cowan University, Joondalup, Western Australia Australia; 5 School of Ocean Sciences, Bangor University, Menai Bridge, UK; 6 Centro de Investigación en Ciencias del Mar y Limnología (CIMAR), Universidad de Costa Rica, San José, Costa Rica; 7 Smithsonian Environmental Research Center, Edgewater, MD USA; 8 Smithsonian Tropical Research Institute, Balboa Ancón, Panama; 9 CIEnAm, Universidade Federal da Bahia, Bahia, Brazil; 10 IAEA Marine Environment Laboratories, Department of Nuclear Sciences and Applications, International Atomic Energy Agency, Monaco, Principality of Monaco; 11 Centre for Tropical Water and Aquatic Ecosystem Research (TropWATER), James Cook University, Townsville, Queensland Australia; 12 Coastal and Estuarine Environment Research Group, Port and Airport Research Institute, Yokosuka, Japan; 13 IHCantabria - Instituto de Hidráulica Ambiental de la Universidad de Cantabria, Santander, Spain; 14 Department of Ecoscience, Aarhus University, Århus, Denmark; 15 Institute for Coastal and Marine Research and Department of Botany, Nelson Mandela University, Gqeberha, South Africa; 16 Centro Agronómico Tropical de Investigación y Enseñanza, Turrialba, Costa Rica; 17 Departament de Física, Universitat Autònoma de Barcelona, Bellaterra, Spain; 18 Excellence Center for Biodiversity of Peninsular Thailand, Faculty of Science, Prince of Songkla University, Hat Yai, Thailand; 19 Dugong and Seagrass Research Station, Faculty of Science, Prince of Songkla University, Hat Yai, Thailand; 20 Intergovernmental Oceanographic Commission of UNESCO, Paris, France; 21 Plymouth Marine Laboratory, Plymouth, UK; 22 Faculty of Environment, Science and Economy, University of Exeter, Exeter, UK; 23 State Key Laboratory of Marine Environmental Science, Key Laboratory of the Ministry of Education for Coastal and Wetland Ecosystems, College of the Environment and Ecology, Xiamen University, Xiamen, China; 24 Centro de Investigación y de Estudios Avanzados de México, Unidad Merida, Merida, Mexico; 25 Institute for Marine and Antarctic Studies, University of Tasmania, Battery Point, Tasmania Australia; 26 Institute of Marine Sciences, University of Dar es Salaam, Dar es Salaam, Tanzania; 27 Louisiana Universities Marine Consortium (LUMCON), Chauvin, LA USA; 28 Scottish Association for Marine Science, Oban, UK

**Keywords:** Scientific community, Carbon cycle, Climate sciences, Environmental social sciences, Ecology

## Abstract

Blue carbon ecosystems, classically defined as mangroves, tidal marshes and seagrasses, but increasingly expanded to include ecosystems such as tidal flats, macroalgal forests and shelf sediments, contribute to climate change mitigation and biodiversity support. Here, seven years after the last global assessment of research priorities, we conducted a priority-setting exercise to identify persistent knowledge and implementation gaps, and the strategic priorities that must be addressed to enable scalable, high-integrity and equitable management of blue carbon ecosystems in a rapidly evolving policy and finance landscape. The highest priority focuses on managing blue carbon ecosystems to support coastal communities while integrating traditional ecological knowledge, emphasizing the essential role of social legitimacy and equity in enabling scalable, long-lasting outcomes. Additional priorities focus on developing cost-effective restoration methods, improving the accuracy of greenhouse gas flux estimates, quantifying the impacts of human activities on carbon cycling and integrating co-benefits such as biodiversity and coastal protection into natural capital frameworks. Emerging technologies like remote sensing, machine learning and data-sharing platforms are also highlighted as transformative tools to fill knowledge gaps and scale solutions. Collectively, these priorities highlight the complexity of blue carbon science and the need for inclusive interdisciplinary approaches that support the resilience and livelihoods of coastal communities.

## Abstract (web summary)

This paper conducted a priority-setting exercise to identify ten questions that define the future direction of blue carbon science. It highlights key gaps, emerging challenges and opportunities for advancing climate mitigation, ecosystem management and evidence-based policy.

## Main

The term ‘blue carbon’ has transitioned from a scientific concept to a formal component of climate policy. Introduced in 2009, it initially referred to the carbon captured and stored by rooted coastal vegetated ecosystems, including mangroves, tidal marshes and seagrasses, characterized by high organic carbon accumulation rates and large, persistent soil carbon stocks^1^. Since then, research and policy have increasingly focused on the manageability of these three blue carbon ecosystems (BCEs), particularly how their conservation and restoration can support climate mitigation by enhancing carbon capture and avoiding emissions from their degradation or loss, while also delivering adaptation co-benefits such as coastal protection and biodiversity support^2,3^. With growing data availability, the scope of blue carbon science has broadened to consider additional ‘emerging’ BCEs (for example, macroalgal forests and tidal flats) with potential relevance for climate mitigation^4,5^.

More recently, the global quantification of carbon stocks and fluxes has enabled their integration into climate mitigation policies, including nationally determined contributions, greenhouse gas (GHG) inventories and carbon markets^6–10^. This policy uptake has generated substantial global interest and accelerated research activity, creating a dynamic feedback between science, policy and implementation.

In 2019, a foundational roadmap identified ten priority questions to strengthen the scientific basis of BCEs as a nature-based solution for climate change mitigation^11^. Since then, substantial scientific advances have been made^12–15^, yet understanding of fundamental processes, such as drivers of carbon accumulation and post-disturbance emissions, remains incomplete. Furthermore, BCE degradation continues to outpace recovery^2,16,17^, and while the complexities of integrating BCE management into mitigation efforts are now better understood^18,19^, targeted research is needed to maximize benefits and address limitations^20–22^.

The policy landscape has also evolved, further elevating the profile of BCEs. International agreements, like the Kunming–Montreal Global Biodiversity Framework, formally recognize blue carbon capture as a critical co-benefit of protecting and restoring BCEs for biodiversity outcomes^23^. Estimates suggest that, under optimal conditions, BCEs could offset 1–3% of global anthropogenic emissions^2,23,24^. While global decarbonization remains essential, the long-term protection and restoration of BCEs can provide a meaningful, domestically controllable mitigation contribution in countries with extensive coastal wetlands, substantial land-sector emissions and important restoration potential^25^.

Given the rapid research growth, evolving policy needs and pressure to deliver outcomes, it is timely to revisit and recalibrate the original roadmap. Following established horizon-scanning and priority-setting frameworks^26,27^, we identified, ranked and refined a new set of priority questions. Rather than repeating earlier exercises, this reassessment examines the most pressing scientific and implementation challenges to enable scalable, credible and equitable conservation and restoration of BCEs, and is intended to guide researchers, practitioners and decision-makers seeking to realize their full potential for climate mitigation, adaptation, biodiversity and coastal livelihoods.

## Priority questions

**Fig. 1:**
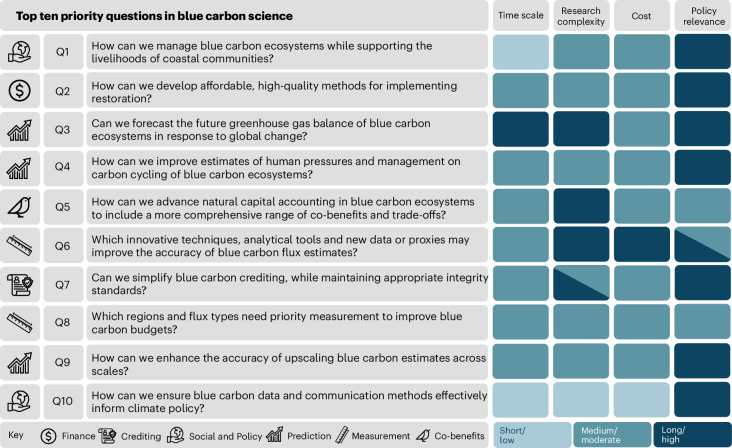
Top ten research priority questions for scalable, high-integrity management of BCEs. The table lists each question alongside its associated theme: finance; crediting; social and policy; prediction; measurement and co-benefits. Each question was assessed across four dimensions (timescale, cost, research complexity and policy relevance) using a three-tier classification system: low (light blue), medium (blue) and high (dark blue). Colours indicate the tier selected by the majority of experts (modal response). Where no single mode emerged (for example, tiers between medium and high), both tiers are displayed. See Table 1 for definitions of each dimension and tier.

The top ten priority questions, identified and ranked by 28 global experts, highlight a field striving to balance technical rigour with practical implementation to ensure credible and scalable blue carbon solutions (Fig. 1). From an initial pool of 116 submissions, the highest-ranked question (Q1) stands as a cornerstone, articulating the challenge of managing BCEs at scale while sustaining coastal livelihoods. Half of the questions (Q3, Q5, Q6, Q7, Q8) focus on strengthening the precision, comparability and scalability of carbon data, underscoring the need for robust evidence to underpin policy development and market mechanisms. The remaining questions (Q2, Q4, Q9, Q10, together with Q1) address the enabling conditions for effective blue carbon governance and finance, including restoration methods, natural capital accounting, crediting standards and science communication. Notably, most questions were rated as highly policy-relevant (Fig. 1; see Table 1 for definitions of each dimension and tier) and considered achievable within a 3–5-year timeframe at moderate cost (US$500,000–2 million) and research complexity, often requiring specialized techniques.

**Table 1:** Classification system used to evaluate the top ten research questions (Q) across four dimensions: (1) timescale, (2) research complexity, (3) cost and (4) policy relevance. Each category is evaluated using a three-tier system (low, medium and high) to guide consistent scoring across expert evaluations while highlighting practical considerations for decision-makers

	Timescale	Data collection complexity	Cost (US$)	Policy relevance
**Classification**	What is the expected timeframe to answer the Q?	How complex is the data collection required to answer the Q?	What is the estimated cost to answer the Q?	How much will answering the Q influence policy?
**Short/simple/low**	1–3 years	Basic data collection methods with minimal technical requirements, such as desk-based analysis of published data or citizen science	<US$500,000; funding from regional agencies, usually from existing budgets or small grants	Data contributes to scientific knowledge but has no clear or direct link to policy
**Medium/moderate**	3–5 years	Requires some specialized techniques or equipment, including routine analyses such as soil organic content, geographic information system mapping and remote sensing	US$500,000–2 million; grants from national organizations	Data aligns with an existing policy framework, although it was not explicitly commissioned or required by policymakers. It may inform policy at regional or local level
**Long/complex/high**	5+ years	Involves advanced methods, specialized equipment and multidisciplinary expertise (for example, environmental DNA analysis, mesocosms for in-situ studies, deployment of advanced sensors)	>US$2 million; multi-partner funding or major global investment	Research is strategically designed to address specific policy needs. It is expected to directly impact national or international policies

### Q1. How can we manage BCEs while supporting the livelihoods of coastal communities?

BCEs and their coastal stewards are deeply interconnected. Management strategies that incorporate local knowledge enhance effectiveness, ensure sustainability and create mutually beneficial outcomes. Early research in marine conservation and biodiversity highlighted the limitations of top-down approaches and underscored the importance of community engagement for conservation success^28^. In response, the integration of local (or traditional) ecological knowledge, rooted in generations of direct interaction with BCEs, has been increasingly acknowledged as a means to improve research and management outcomes^28,29^. Fiji’s locally managed marine areas are frequently cited as an example of participatory management supporting both conservation objectives and sustainable livelihoods^30^. Although these arrangements have strengthened community engagement and local governance, recent analyses suggest that they do not necessarily result in clear ecological or socio-economic gains^31^. This highlights the complexity of linking conservation interventions with measurable outcomes and the importance of new resources, such as *Including Local Ecological Knowledge (LEK) in Mangrove Conservation & Restoration*^32^, which offers practical guidance and case studies for the ethical integration of local and traditional ecological knowledge into research and project design.

**Fig. 2:**
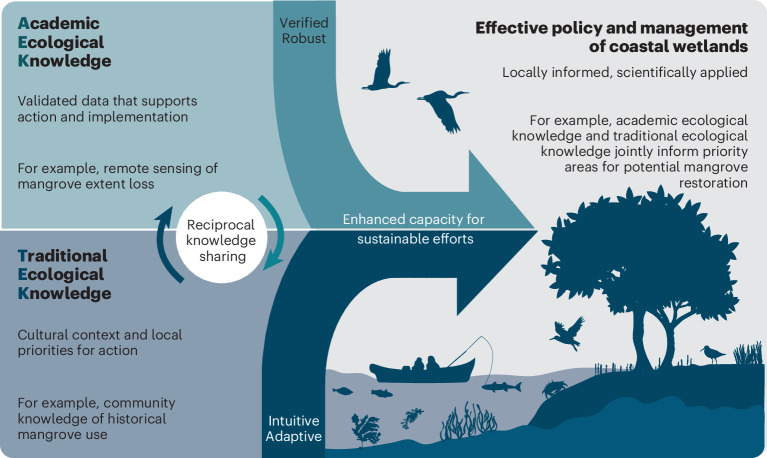
Reciprocal knowledge sharing in BCE management. Visual representation of the continuous exchange between ‘academic ecological knowledge’ and ‘traditional ecological knowledge’, highlighting how their combined insights contribute to more effective and sustainable management efforts. Adapted from ref. ^29^, Springer Science & Business Media.

To ensure both the long-term persistence of BCEs and the livelihoods they support, future conservation and management must integrate local and traditional ecological knowledge with scientific research, also known as academic ecological knowledge. This fusion enables the refinement of best practices within a modern context^29,33^ (Fig. 2). Sustainable BCE conservation depends on knowledge sharing, capacity building and inclusive approaches that prioritize local needs and participation. Achieving this requires moving away from one-size-fits-all management models (for example, blanket no-take marine protected area) and ensuring that research and funding deliver tangible benefits to local communities rather than external stakeholders.

Conservation efforts should recognize and manage the full range of ecosystem services BCEs provide^34^, particularly those that directly underpin coastal livelihoods and deliver tangible, recurring benefits. The sustainable use of BCEs will vary across locations, as the relative importance of ecosystem services can vary according to factors such as coastal geomorphology and cultural practices. Transparency in blue carbon projects is essential to ensure that efforts support ecosystem health while promoting more equitable livelihoods^35^. Poorly designed projects can inadvertently worsen inequities if they fail to account for local socio-economic dynamics. While traditional ecological knowledge-based conservation inherently adopts a holistic approach to managing entire watersheds^28^, this perspective is not consistently reflected in research-driven management plans or blue carbon project development. Landscape-scale approaches, such as ‘ridge to reef’ or ‘coastal corridors’, offer valuable opportunities to connect ecosystems and land-use categories beyond man-made boundaries.

Investments in blue carbon projects should align with community priorities, available coastal resources and varying government actions to ensure holistic and equitable outcomes^34^. Priority areas for blue carbon projects should not be solely determined by resource (ecosystem) availability. Even resource-rich regions may face lower conservation success if investments and development plans are inequitable, harmful or fail to integrate local priorities. Sustainability in BCE management and equitable participation in the blue economy rely on understanding and respecting the stewardship practices of local communities and Indigenous peoples, ensuring that conservation efforts are culturally appropriate, mutually beneficial and contribute to long-term environmental and socio-economic resilience^29,36^.

### Q2. How can we develop affordable, high-quality methods for implementing restoration?

Effective recovery of BCEs requires first addressing the drivers of ecosystem decline, which should always precede any active restoration efforts^37,38^. Once the ecological conditions for restoration are in place, targeted active restoration may accelerate BCE recovery. Testing and refining low-cost, effective restoration approaches is essential to increase the likelihood of restoring ecosystem structure and function, including carbon storage.

Despite growing interest, the costs, successes and failures of restoration projects are often underreported^39–41^. Restoration costs strongly influence project feasibility and scalability and vary widely across countries, with lower costs in the global south (that is, regions with typically lower incomes and research capacity) reflecting reduced labour expenses^39^. Costs also depend on both methods and scale. While larger projects often achieve lower costs per hectare^39^, restoration success strongly depends on the recovery of site-specific ecological condition and function, rather than the scale of investment. Among BCEs, mangrove restoration is the least expensive (median: US$9,000 ha^−1^)^39,42^, while seagrasses, tidal marshes and emerging BCEs (for example, macroalgae) can be substantially more costly to restore^39^.

Mangrove restoration has traditionally relied on planting, but this is now discouraged as a primary strategy and is instead used to support natural regeneration where hydrology, elevation and soil quality are suitable^43^. Ecological mangrove restoration is now preferred, as it restores tidal flows and biophysical conditions to promote natural regeneration^44^. This approach supports faster biodiversity recovery^45^ and costs are comparable to planting, with planting averaging US$1,191 ha^−1^ (ref. ^39^) in the global south, and ecological restoration averaging US$1,388 ha^−1^ in Indonesia^45^. Final costs vary with seedling price, land preparation, permitting and monitoring effort.

Seagrass active restoration (for example, transplantation or seeding) is typically undertaken when natural recolonization is limited by propagule supply. These methods can accelerate recovery, but success is still highly variable and often constrained by scale and cost^37,46,47^. Outcomes depend on method, labour and local environmental conditions^39^, with larger-scale interventions generally performing better^37^. Methods effective in one region may fail elsewhere due to differences in species life histories, structural traits and functional roles^48,49^. Most methodologies have been developed in wealthier nations, where reproductive strategies (for example, flowering and seed production) are well understood^50,51^. Combined with high costs, low success rates (11% in the global south versus higher elsewhere) and inadequate long-term support, knowledge gaps restrict the scaling of seagrass restoration worldwide^39^.

Tidal marsh restoration is typically achieved through managed realignment, hydrological reconnection or restoring tidal elevation^52^. Costs vary widely with method and context: global syntheses estimate US$9,000–90,000 ha^−1^, with a mean of ~US$38,000 ha^−1^ (ref. ^39^). More recent reviews suggest median costs of ~US$24,000 ha^−1^, though projects involving major earthworks or land purchase can exceed US$200,000 ha^−1^ (ref. ^53^). Outcomes vary substantially, with some sites regaining vegetation and carbon burial rates within 5–10 years^54^, while others take decades depending on sediment supply, tidal range and grazing pressure^55^.

Delivering cost-effective, high-quality BCE restoration depends on local economic and logistical context, the degree of degradation and the suitability of restoration approaches. Costs can be reduced by involving local communities and volunteers, and by developing adaptive techniques using locally available materials and species^56,57^. Pre-feasibility assessments that identify drivers of degradation can guide targeted site-specific restoration actions, while building local capacity for monitoring, reporting and verification (MRV) is also essential for long-term success.

### Q3. Can we forecast the future GHG balance of BCEs in response to global change?

Forecasting the GHG balance of BCEs under changing conditions is essential for carbon financing and other activities that rely on predicting the permanence of sequestered carbon and continued atmospheric CO_2_ removal. This is particularly challenging under anthropogenic pressures such as climate change, which strongly affect GHG budgets. While reference sites can provide implicit forecasts for restoration goals, numerical models are needed to represent key mechanisms and processes across diverse hydrogeomorphic settings (distinct from models that assess the GHG effects of human impacts in Q4). Accurate predictions also require high-quality data on organic carbon accumulation (or net CO_2_ exchange), nitrogen cycling^48^ and GHG fluxes^49^ across global biogeographic zones and diverse coastal environmental settings (Q8). Understanding how carbon dynamics respond to biophysical, chemical and environmental drivers (for example, nutrients/sediments, salinity, temperature, sea-level-driven accommodation space), including their relative importance, interactions and timescales, is also crucial as carbon accumulation rates and GHG fluxes are influenced by these factors.

Several reviews have focused on predicting changes in the spatial distribution of BCEs, organic carbon accumulation and stocks under anthropogenic and climate change scenarios^11,58–61^. For instance, sea-level rise is projected to drive coastal squeeze in tidal marshes and other coastal wetlands, reducing habitat space and limiting carbon storage and accumulation potential^62,63^. This growing mechanistic understanding has improved forecasting of organic carbon accumulation and habitat distribution, providing proxies for evaluating BCE climate mitigation potential^64^ and informing accredited blue carbon methodologies, some of which incorporate risk assessments for stock losses due to factors such as project management, land tenure or extreme weather events^65^.

Advances in mechanistic understanding have enhanced process-based models, which, due to their reliance on well-understood relationships between GHG fluxes, organic carbon accumulation and drivers, are valuable tools for forecasting GHG balance under changing environmental conditions or modifications. Several soil cohort models designed for tidal marshes and mangroves predict organic matter and carbon accumulation, as well as sediment accretion, with some explicitly simulating responses to sea level rise^66–70^. The next step is expanding these models to predict the GHG balance, incorporating CH_4_ and N_2_O fluxes, as well as lateral exchanges of dissolved GHGs and carbon across habitats (Q6, Q8)^71,72^.

Unlike in terrestrial wetlands, comprehensive process-based models for forecasting emissions under changing environmental conditions remain underdeveloped in BCEs. Some progress exists, such as the PEPRMT-tidal model for marsh ecosystems^73^, which predicts CO_2_ and CH_4_ emissions and carbon accumulation by coupling with the cohort marsh equilibrium model^69^, and the denitrification–decomposition model, which simulates carbon and nitrogen dynamics in mangroves^74^ and tidal marshes^75^, providing estimates of biomass, soil carbon and GHG fluxes. However, models that incorporate lateral carbon exchange in all BCEs (Q6) or forecast GHG budgets beyond soil and biomass stock changes for submerged aquatic vegetation (for example, seagrasses and macroalgae) still need to be developed. Application beyond classical BCEs is also critical to pace science advancements in additional coastal wetlands^76^. Lateral carbon transport is important for the GHG balance, particularly in habitats with submerged aquatic vegetation, but the impact of climate-change-induced changes in ocean hydraulic structure (for example, currents and stratification) and air–water gas re-equilibrium on GHG balance remains unknown^77^.

### Q4. How can we improve estimates of human pressures and management on carbon cycling of BCEs?

**Fig. 3:**
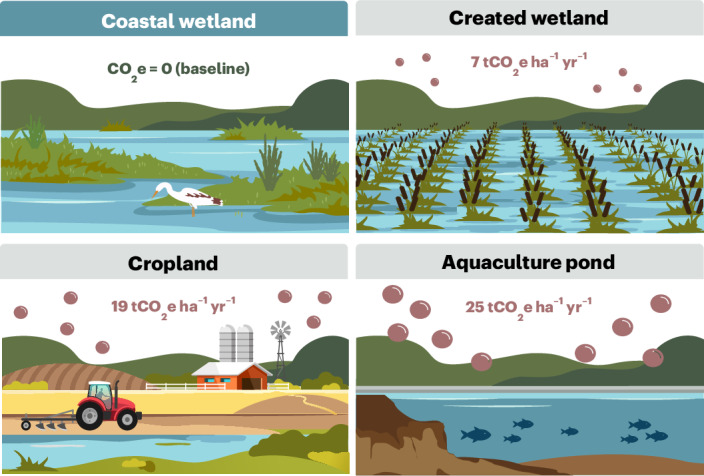
LULUC impacts on coastal wetland GHG emissions. Converting natural coastal wetlands to freshwater wetlands, cropland or aquaculture ponds increases GHG emissions to 7, 19 and 25 tCO_2_e ha^−1^ yr^−1^, respectively. Reversing such changes through restoration has the potential to lower GHG emissions. Values represent net radiative forcing from CO_2_, CH_4_ and N_2_O combined, with natural wetlands as the baseline (net emissions ≈ 0). Data are from a meta-analysis in ref. ^78^, the most comprehensive to date, though still constrained by limited sample sizes (<12 studies for key variables, particularly in converted wetlands). Bubble sizes are illustrative and not to scale.

Human activities in BCEs generally increase net GHG emissions that contribute to radiative forcing^78^ (Fig. 3). These emissions create both challenges and opportunities; while degradation accelerates emissions, protecting and restoring these ecosystems can support climate mitigation and deliver multiple co-benefits. Realizing these benefits requires robust data collection and analysis to quantify baseline conditions and project GHG reductions. Limited access to data, analytical tools and measurement technologies at the necessary spatial and temporal scales remains a major barrier to advancing blue carbon science and applications.

Despite important advancements over the past decade, critical knowledge gaps persist in assessing the effectiveness of BCEs for GHG mitigation. Soil and biomass carbon pools are among the best-constrained parameters, having been synthesized across multiple spatial scales and incorporated into coherent databases^79–82^. However, coverage remains uneven, particularly for seagrasses and BCEs in the global south^79,83^. Existing maps for upscaling point data to broader seascape units are relatively reliable for mangroves, but remain less developed or unavailable for other BCEs, limiting verification of their effectiveness in carbon accumulation and refinement of global estimates. Current datasets are biased towards intact ecosystems, with limited information on how land use and land-use change (LULUC) and forestry influence carbon dynamics, particularly following disturbance or restoration.

A major challenge in blue carbon inventories is the scarcity of data on carbon and GHG fluxes (Q8), particularly CH_4_ and N_2_O, which have high global warming potential and introduce the largest uncertainty into estimates of LULUC and forestry effects on radiative forcing^84^. Syntheses of chamber-based and eddy flux data have improved organic carbon budgets at continental and global scales^85,86^, yet remain insufficient for quantifying emissions at small-project scales where field measurements are not feasible.

While continued research on the mechanisms driving soil carbon accumulation is essential^87^ (Q3, Q8, Q9), priority should also be given to developing flux-relevant proxies for these processes, including local sea-level rise^88^, geomorphic setting^81^, vegetation structure and productivity^89^, and suspended sediment^90^. These processes are partially captured in robust numerical models that predict tidal marsh elevation changes in response to sea-level rise^90^, warming^91^ and elevated CO_2_ (ref. ^92^). However, existing models mainly apply to tidal marshes and mangroves, without being directly transferable to other BCEs with woody vegetation (that is, tidal freshwater forested wetlands) or coastal plants (that is, seagrasses), limiting their applicability for forecasting LULUC and restoration impacts on radiative forcing across BCEs.

The basic processes governing CH_4_ and N_2_O emissions are well known, but our ability to model their spatial and temporal variability remains limited. Salinity is a strong predictor of CH₄ emissions at broad spatial scales^85^, but local variations often depend on additional factors, such as distance to tidal creeks, plant traits and microbial community composition^93–95^. Data on N_2_O flux drivers are even more limited, though emissions tend to be low in the absence of external nitrate loading^96^. This could change as high-intensity agriculture continues to expand into BCE-adjacent areas, increasing nutrient inputs and potentially altering emission dynamics. Least understood are the processes that govern hydrologic fluxes of organic carbon and GHGs, the consequences of LULUC on these fluxes and the fate of exported compounds in adjacent marine ecosystems.

Quantifying the effects of LULUC on BCEs is scientifically and technically challenging, requiring sustained investment in research. Future efforts should focus on ecosystem features that can be remotely sensed and integrated with data from sensor networks, empirical measurements and scale-appropriate models, to produce high-resolution maps for applications ranging from site-level restoration projects to national inventories.

Progress will be greatly accelerated by improved mapping capability and open-access platforms for trusted data sharing that support MRV (for example, ref. ^79^), particularly where remote-sensing data from managed networks is integrated with flux-relevant proxies and numerical models^97^. Data sharing should allow information to be easy to find, accessible, interoperable across systems and reusable^98^, particularly for underrepresented and emerging BCEs. Addressing these challenges will be essential for fully integrating BCEs into global climate strategies.

### Q5. How can we advance natural capital accounting in BCEs to include a more comprehensive range of co-benefits and trade-offs?

Understanding the full range of co-benefits and trade-offs in natural capital accounting for BCEs requires a robust framework that integrates ecosystem dynamics, service valuation and long-term monitoring. The System of Environmental-Economic Accounting (SEEA) is the international standard for quantifying spatial and temporal relationships and dynamics between ecosystem extent, condition, services provided and economic value^99^. SEEA informs economic and environmental policies^100^, business accounting^101^ and multiple global conventions. It typically focuses on individual environmental components such as carbon, water or biodiversity, which can be aggregated from local to national and global natural capital accounts^100^. For instance, in ref. ^102^ the authors use country-specific social costs of carbon to estimate that BCEs contribute US$190.67 billion per year in global wealth.

To fully capture the co-benefits and trade-offs of BCEs, SEEA frameworks require coordinated assessments, stakeholder engagement, clear institutional mandates and sustained resources for data collection and MRV. A key challenge is transitioning from valuing individual ecosystem services to aggregating them at the ecosystem level, given the complex assessment requirements for doing so^103^. Clear guidance on how to achieve this is needed to harmonize data across countries, alongside strengthened technical capacity in natural capital accounting, ecosystem valuation and sustainable management, particularly in the global south.

Australia recently developed a guide for applying the SEEA framework to BCEs, detailing the methodologies for assessing restoration benefits^104^. The guide outlines approaches to measuring and valuing various ecosystem services, including carbon accumulation, water purification, coastal protection and cultural services, with example SEEA-aligned tables for tracking ecosystem changes due to restoration. Its application is demonstrated in the Hunter River estuary in New South Wales and East Trinity Inlet in Queensland. In the former, restoration efforts improved tidal marsh and supratidal forest ecosystems, leading to increased biomass, benefits to fisheries and recreation, and carbon abatement through avoided emissions and enhanced accumulation^105^. In the latter, restoration reduced acid sulfate soil impacts, improved water quality, expanded mangrove and tidal marsh areas, and strengthened ecosystem connectivity. Cultural services for the Mandingalbay Yidinji people were also incorporated^106^.

Despite this substantial progress, underrepresented and emerging BCEs remain excluded from global frameworks, markets and natural capital accounting^107^. Expanding research to verify their effectiveness in delivering a wide range of ecosystem services will be critical for refining natural capital accounts and ensuring that BCE co-benefits and trade-offs are accurately represented. Practical management techniques and frameworks are also needed to facilitate their inclusion in conservation and climate strategies. As financial interest in blue carbon accounting grows (estimated at US$10 billion or more)^108^, aligning ecosystem service benefits with funding mechanisms may help support informed, equitable and actionable decisions regarding sustainable development, climate adaptation and BCE conservation.

### Q6. Which innovative techniques, analytical tools and new data or proxies may improve the accuracy of blue carbon flux estimates?

Quantifying blue carbon requires an integrated approach that combines remote sensing, in-situ measurements of above- and below-ground biomass and soil organic carbon, and machine learning techniques, ideally encompassing both stores and flows of dissolved and particulate organic and inorganic carbon, as well as the associated gas fluxes. These fluxes occur vertically and laterally, driven by natural biogeochemical processes within coastal ecosystems and their adjacent environments. Although vertical and lateral carbon fluxes can be substantial in some BCEs, their high spatial and temporal variability makes them difficult to quantify (see Q4 for a discussion of these constraints).

The traditional approach to estimating the organic carbon density of BCEs (that is, soil organic carbon, below- and above-ground biomass per unit area) relies on point-based field sampling, sediment coring and laboratory analysis of biomass organic carbon and soil organic carbon, often combined with sediment dating to assess long-term carbon accumulation. While highly accurate, this approach is time-intensive, costly and limited in spatial coverage. To overcome these limitations, integrating remote sensing with in-situ measurements and new machine learning techniques offers a promising, scalable and cost-effective alternative for mapping carbon stocks and fluxes across BCEs^109,110^. However, remote sensing alone cannot estimate soil organic carbon accumulation rates, which determine the long-term accumulation of atmospheric carbon. The high spatial and temporal variability of these accumulation rates, even within a single BCE, further constrains large-scale extrapolation^20,83^.

A growing suite of in-situ sensors and flux networks enables continuous, multi-scale observation of CO_2_, CH_4_ and N_2_O in BCEs, such as eddy-covariance systems coupled to infrared or laser spectrometers^111^. Eddy-covariance methods from atmospheric science are increasingly adapted to tidal marshes and mangroves, where combining tower fluxes with burial and lateral exchanges can close the net ecosystem carbon balance^112,113^. Practical guidance now emphasizes gas-specific method selection, chamber design and deployment frequency, along with quality assurance and control procedures to reduce bias^114^. For stock and emission-factor work, standardized field protocols remain essential for comparability across mangroves, tidal marshes and seagrasses^115^. Critically, non-CO_2_ gases can alter net climate benefit (for example, seagrass CH_4_ can reduce, whereas N_2_O dynamics can enhance apparent sinks), so integrated GHG measurement is required^116^. Recent guidance calls for standardized protocols, transparent uncertainty analysis and long-term distributed observatories to support credible MRV and policy uptake^114^.

Recent advances in remote sensing, including multispectral, hyperspectral and synthetic aperture radar imagery, generate rich spectral, spatial and multi-temporal data on BCEs^117^. As these sensors capture complementary structural attributes of BCEs, machine learning approaches that integrate multimodal Earth observations through data fusion models are increasingly important for scaling of carbon stocks and fluxes^110^. Cloud computing platforms, such as the Google Earth Engine, further support scalable processing of large remote-sensing datasets^110,118^, and species-level classification, ensemble-based decision trees and deep learning approaches have proven effective for improving retrieval accuracy and tracking carbon changes over time^110^. Continued advances in remote sensing and artificial intelligence, alongside collaboration between researchers, policymakers and stakeholders, will be critical for refining global BCE carbon assessments and supporting investment in blue carbon projects^110^. Ensuring equitable access to these technologies through capacity building and training, particularly in regions with extensive BCEs, is essential for reducing data disparities between the global south and the global north.

### Q7. Can we simplify blue carbon crediting, while maintaining appropriate integrity standards?

Although historically rates of BCE loss have declined, total area loss still outpaces restoration and creation efforts^17,119,120^. Carbon financing could support conservation, restoration and creation of BCEs, but project uptake remains low due to numerous technical, financial and social barriers^108,121^. A major challenge is the complexity of quantifying organic carbon stocks and fluxes, which requires specialized expertise and evidential support. While several methodologies exist to simplify carbon accounting, most remain too complex or costly for widespread community implementation. This raises the question of whether blue carbon crediting methodologies can be further simplified without compromising scientific rigour.

Such simplification may be feasible without compromising project integrity and standards. Existing frameworks, including Verra and Plan Vivo, could streamline monitoring protocols by leveraging wider data availability or adopting tiered verification procedures similar to the Intergovernmental Panel on Climate Change’s (IPCC) tier system. The key challenge is linking the primary drivers of organic carbon accumulation and the magnitude of GHG fluxes, ensuring that underlying assumptions are well supported by empirical data. Developing reliable default values at specific spatial scales requires high-quality datasets that capture diverse geomorphic, hydrological, hydrodynamic and ecological conditions, including species composition and stand structure. For some BCEs, such as mangroves, existing datasets on organic carbon stocks and GHG fluxes provide representative default values for national, regional or species-specific baseline assessments^122–124^ and support high-quality models to estimate stocks and fluxes under different management scenarios^8,12^. However, such models may not fully capture site-specific mechanisms driving blue carbon dynamics. The treatment of allochthonous organic carbon also remains a critical challenge for assessing additionality, underscoring the need for robust observational and experimental approaches to support blue carbon crediting frameworks^125^.

Recent progress includes the synthesis of high-quality datasets and the development of comprehensive databases, such as the Coastal Carbon Library and Atlas^79^ and the EURO-CARBON database^126^. These resources provide baseline reference data and highlight underrepresented BCEs and regions with data deficiencies^127^. However, many regions still lack the capacity to generate the high-quality datasets needed to improve the accuracy and inclusivity of carbon accounting^82,128^. Building capacity through global and regional training centres, research hubs and context-appropriate methodologies could help bridge these gaps by enhancing technical expertise, facilitating the development of spatially unbiased models, and establishing robust baseline carbon stock and flux values. These efforts would ultimately support the broader adoption of blue carbon crediting and strengthen the representation of BCEs in global carbon markets.

### Q8. Which regions and flux types need priority measurement to improve blue carbon budgets?

Most BCE research has focused on quantifying carbon stocks in soils and biomass^79,80^, with comparatively fewer studies addressing fluxes^72,86^, despite their importance for understanding net carbon balance. This gap is particularly acute in restored BCEs, where limited comparisons of carbon fluxes with reference sites constrain assessments of restoration additionality (but see ref. ^129^).

**Fig. 4:**
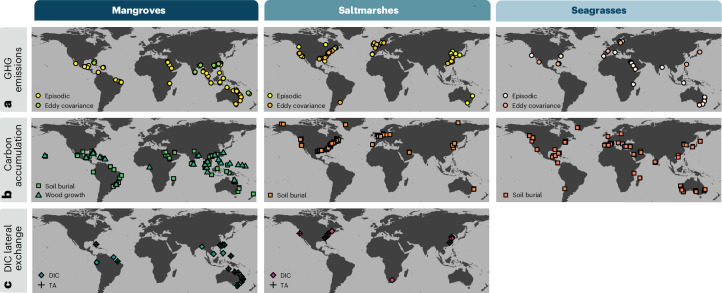
Global distribution of carbon flux datasets for mangroves, tidal marshes and seagrasses. **a**, GHG emissions (GHGs: CO_2_, CH_4_ and N_2_O), derived from continuous eddy covariance and episodic data from chamber, headspace equilibration or seawater–air exchange (data from refs. ^86,116^). **b**, Carbon accumulation in BCE soils and mangrove woody biomass (compiled data from refs. ^139,166,167^). **c**, Lateral exchange of dissolved inorganic carbon (DIC) and total alkalinity (TA) (data from ref. ^72^).

Long-term monitoring systems that integrate local- and national-scale data are essential, yet improving carbon flux estimates is constrained by the logistical and financial burden of long-term monitoring, particularly for highly variable ecosystem-scale GHG fluxes. Techniques such as eddy covariance flux towers provide valuable continuous measurements but are expensive and rarely deployed across BCEs. In addition, data on lateral carbon exchange remain sparse, particularly for particulate organic carbon, dissolved oganic and inorganic carbon, and total alkalinity export. These lateral fluxes, especially total alkalinity export, may account for 25–40% of the carbon budgets in mangroves and tidal marshes^72,130^. Although carbon fluxes are needed for conservative estimates of carbon uptake and long-term removal, they are more site-specific than carbon stocks and, therefore, poorly represent the global diversity of coastal geomorphic and climate settings where BCEs occur (Fig. 4). Accounting for timescales is also critical, as they affect estimates of organic carbon preservation^21,22,131^. Addressing many of these data gaps would benefit from new protocols to estimate lateral carbon fluxes^132^ and sustained investment in global-scale monitoring networks.

The availability and distribution of GHG flux measurements vary substantially across regions and BCEs (Fig. 4). Mangrove and seagrass flux estimates largely rely on episodic measurements (for example, chambers, headspace equilibration, seawater–air exchange), whereas tidal marshes are more frequently monitored with eddy covariance flux systems that provide higher temporal resolution of net ecosystem exchange. However, monitoring efforts are heavily concentrated in the global north, particularly in subtropical and temperate regions, resulting in notable gaps for tropical tidal marshes and seagrasses (Fig. 4), as well as Nordic/Baltic, subarctic and arctic BCEs^133–135^. Likewise, while soil organic carbon accumulation rates (based on ^210^Pb and/or ^137^Cs) are relatively well-documented in mangroves and seagrasses, data for tropical tidal marshes^136^ and emergent BCEs are largely missing.

Efforts to characterize the ecological and geomorphic drivers of BCE carbon dynamics across contrasting geographies have advanced, yet critical gaps persist^60^. While key environmental drivers of carbon stocks in seagrasses and carbon accumulation in mangroves have been identified across coastal geomorphic settings (for example, river-dominated to carbonate coastlines)^14,81,137–139^, a unified typology spanning multiple BCEs would improve comparability and predictive modelling. Species composition strongly influences seagrass carbon stocks^14^, and global patterns in tidal marsh soil organic carbon stocks are emerging^140^. In contrast, the role of coastal geomorphic settings on GHG emissions^86^ and lateral carbon exchange remains poorly understood^72,141^, with limited observations preventing clear global patterns. Addressing these data gaps requires an internationally coordinated effort to establish long-term observatory networks across diverse climate zones and coastal geomorphic settings, enabling conservative estimates of net ecosystem carbon balance. Such monitoring would also improve assessments of BCE services at scale and anticipate future data needs to ensure robust, accurate, site-specific and globally representative blue carbon assessments that support conservation, restoration and climate mitigation strategies. Improved monitoring also enhances the reliability of regional assessments, which directly inform the upscaling of blue carbon estimates across scales (Q9). An additional way to improve estimates of carbon uptake and long-term removal is to integrate BCEs into national GHG inventories, following the IPCC’s wetlands supplement^6^, as demonstrated in Australia, Costa Rica and the USA.

### Q9. How can we enhance the accuracy of upscaling blue carbon estimates across scales?

The development of robust methods for collecting and synthesizing observational data has improved global and national assessments of carbon stocks, GHG fluxes and the distribution of BCEs^85,122,142,143^. However, with many regions and BCEs remaining data-limited (Q8), effective upscaling techniques are needed to translate available observations into reliable, scalable estimates while accounting for spatial heterogeneity across scales (Q9). One approach is statistical upscaling, whereby blue carbon features are predicted from geological, hydrological and biogeochemical parameters. Ideally, these models account for spatial heterogeneity from local to regional scales, but identifying predictors that remain consistent across multiple scales remains challenging. Scale-independent processes could offer promising insights by making reliable, generalizable predictions from measured data^110,117^. Observational-based upscaling benefits from using direct measurements of carbon stocks or process rates, which can then be extrapolated. When blue carbon features exhibit nonlinear relationships with multiple predictors, machine learning may offer higher-precision upscaling^64^. In this case, models require comprehensive datasets with complete blue carbon feature values and multiple predictor variables, which are often unavailable. Standardized archiving of datasets across regions is, therefore, essential^79^.

Another approach involves first estimating the spatial extent of BCEs and then upscaling carbon stocks or fluxes accordingly. Traditionally, large-scale estimation of BCE extent has relied on remote sensing, but challenges remain, particularly for submerged ecosystems such as macroalgal forests and seafloor habitats^110^. Expanding high-precision observational techniques and acoustic methods can improve the accuracy of remote-sensing-based BCE estimates^110^ (Q6).

Beyond remote sensing, numerical modelling provides an alternative approach to estimating BCE extent. Such models fall into statistical or mechanistic categories^144^. Statistical models use geo-referenced species observations and environmental parameters to define a multivariate space of suitable environmental conditions, which are then used to parameterize species distributions^143,145–148^. While useful, these models simplify complex ecological processes and can be difficult to interpret, particularly for detecting change over time (that is, inference). In contrast, mechanistic models incorporate species traits (for example, morphology, physiology, demography) to establish direct links between environmental conditions and species distributions^144^. By integrating ecological understanding, mechanistic models offer more robust, long-term and large-scale predictions^149^ and are now beginning to emerge for key BCE species^150,151^.

Upscaling carbon stocks and fluxes can be achieved by estimating BCE extent (via remote sensing or modelling) and multiplying it by a known measured carbon stock value (for example, carbon content) or process rates (for example, carbon accumulation rates)^152^. Alternatively, spatial extent can be coupled with physical models to estimate carbon fluxes linked to BCEs across ocean domains^153^. Distribution maps are available for classical BCEs and (to an extent) macroalgae forests (Table 2), but only local-to-regional data are available for underrepresented or emerging BCEs. Improving spatial coverage across all BCEs (classical and emerging) and updating maps to account for LULUC (Q4) are, therefore, essential^16^.

**Table 2:** Summary of distribution maps available for different BCEs, including data sources and estimated extent

BCE	Area (km^2^)	Data source
Mangroves	137,760–147,359	^161,162^
Tidal flats	124,286–131,821	^163,164^
Seagrasses	160,387–266,562	^143^
Tidal marshes	52,880–54,951	^136,165^
Macroalgal forests^a^	6.06–7.22 million	^147^
Kelp forests	1.47 million	^146^

^a^Includes red algae, green algae and brown algae (kelp).

### Q10. How can we ensure blue carbon data and communication methods effectively inform climate policy?

The term ‘blue carbon’ was initially introduced as a policy and marketing strategy to promote conservation, restoration and management of coastal vegetated ecosystems based on their carbon storage and climate change mitigation potential^1^. Over time, it evolved into a commonly used noun, reflecting its acceptance and integration into scientific and policy discourse. However, communicating carbon stock and flux findings and integrating them into policy remain key challenges.

Policymakers require robust and scientifically credible metrics to inform decision-making^154^, yet the complexity and variability of BCEs, along with data gaps and methodology standardization, have made science translation challenging. Blue carbon science would benefit from coordinated international research and standardized monitoring to address data gaps, particularly in the global south, underrepresented regions and emerging BCEs. International guidelines should be updated and expanded to reflect the latest high-quality data and ensure equitable access to data and methodologies. While mangroves, tidal marshes and seagrasses have been included in the IPCC’s 2014 guidelines^24^, emerging BCEs remain excluded because of insufficient documentation of additionality and permanence of carbon storage (for example, tidal flats, macroalgae forests^4,155^). Uncertainty in carbon accumulation rates and reference values further constrain the development of robust blue carbon accounting frameworks. Hence, the IPCC’s guidelines should be regularly updated using new high-quality data generated through standardized protocols across classical and emerging BCEs. Revising the IPCC’s tier values to reflect the vast increase in data over the past decade would further improve inventory reliability, ensuring a more accurate representation of blue carbon in global climate strategies^9,10^ and carbon markets^121^.

Clear, consistent and concise communication strategies are critical to accurately convey the benefits and limitations of blue carbon to policymakers and the public. Transparent, data-driven messaging builds public trust and reduces misperceptions about the role of BCEs in carbon accumulation and emissions^21^. Equally important is highlighting co-benefits beyond carbon storage, including coastal protection, biodiversity enhancement and nutrient cycling^156,157^, which can support the inclusion of BCEs in climate adaptation strategies. Interdisciplinary collaboration is, therefore, needed to integrate co-benefits and socio-economic considerations into blue carbon strategies^158^.

Closer collaboration with policymakers would enable blue carbon science to more directly inform policy decisions, international agreements and national climate action plans. Global initiatives, such as the United Nations Decade on Ecosystem Restoration (2021–2030) and the Kunming–Montreal Global Biodiversity Framework, offer opportunities to align blue carbon with international policy agendas^159^. However, stronger coordination across conventions like the United Nations Framework Convention on Climate Change, the Convention on Biological Diversity and the Ramsar Convention on Wetlands of International Importance, is needed to enhance policy coherence and implementation^10^. Integrating blue carbon accounting into national climate strategies and expanding its role in carbon markets will require concerted efforts to standardize methodologies and improve data accessibility.

## Outlook and conclusion

Blue carbon science is entering a new phase, with growing demand for evidence to inform climate mitigation, coastal resilience and biodiversity goals. Interest from governments and the private sector, including emerging biodiversity and blue carbon credit mechanisms, has created new opportunities, but also heightened expectations for accuracy, transparency and social legitimacy. Meeting these expectations requires resolving persistent scientific and implementation challenges.

Current approaches for quantifying carbon stocks and GHG fluxes still face major uncertainties across dynamic coastal landscapes, reducing the reliability of carbon accounting frameworks and the credibility of market instruments such as carbon credits and nationally determined contributions. Translating blue carbon science into action, therefore, depends on filling geographic and ecosystem data gaps, improving access to technological tools and strengthening the socio-economic dimensions of BCE management, including community engagement and equitable benefit-sharing.

Comparing the current priority questions to those identified in the 2019 publication ‘The future of blue carbon science’^11^ reveals both continuity and evolution (Supplementary Table 1). Greater emphasis is now placed on community livelihoods, scaling and forecasting challenges, lateral carbon fluxes and policy-ready estimates of carbon accumulation and GHG fluxes. The field has expanded beyond foundational carbon metrics to a more critical evaluation of BCE management interventions, recognizing that conservative, evidence-based estimates are essential for policy relevance^21,22,125^. Priority questions now also address restoration costs, accounting of ecosystem services, organic carbon provenance across seascapes and the role of management actions in shaping long-term carbon outcomes^19,39,104^.

The prioritization of social dimensions, including coastal community implications, co-benefits such as biodiversity support and coastal protection, and improved data sharing and communication, reflects a more holistic framing of BCEs^28,79^. The field has also expanded to encompass additional marine and coastal ecosystems, such as supratidal forests, mudflats and macroalgal forests^4,155^, and the role of lateral carbon fluxes across habitats^72^. As a result, blue carbon research increasingly adapts a more comprehensive and interdisciplinary approach to understanding carbon dynamics across diverse coastal ecosystems^160^.

In summary, blue carbon science has matured into a multidisciplinary field that critically evaluates BCE management^2^. By integrating co-benefits, policy relevance and community well-being, the discipline is increasingly aligned with global priorities in climate change mitigation and ecosystem-based adaptation, coupling scientific rigour with practical pathways for implementation^3,23,36^.

## Methods

A priority-setting exercise was conducted during a workshop held at the International Atomic Energy Agency headquarters in Vienna, Austria (13–16 November 2023), as part of the Global Ocean Decade Programme for Blue Carbon. The workshop convened 28 BCE experts representing academic, governmental and non-governmental institutions from 16 countries across 6 global regions (Australasia, North, Central and South America, Europe, Africa and Asia; see Supplementary Text 1 for country details). Participants reflected balanced gender representation and a wide range of career stages and disciplinary backgrounds, including policy, social science, ocean science, soil science, remote sensing and wetland ecology.

### Question elicitation and prioritization

To identify key research priorities in blue carbon science and policy, we adapted the methodology of ref. ^26^ and incorporated open online voting, similar to the approach used by ref. ^27^. Participants were invited to submit up to five priority questions focusing on actionable challenges in classical and emerging BCEs. Questions were required to be concise, solution-oriented and distinct from existing outputs, with emphasis on novelty, practical relevance and potential impact.

In total, 116 questions were submitted, consolidated to remove duplicates, and organized into 9 thematic categories to support structured group discussions: (a) boundaries and definitions; (b) emerging BCEs; (c) prediction; (d) measurement; (e) crediting and standards; (f) co-benefits; (g) communication; (h) finance and markets; and (i) social and policy (see Supplementary Text 2 for thematic definitions and Supplementary Data 1 for question classification). Participants were then divided into six discussion groups (4–6 experts each), balanced across expertise, geography, gender and career stage, with each group including at least one subject-matter expert and one non-expert. To balance workload, themes (c), (d) and (e) (accounting for ~65% of all submitted questions) were each allocated to a dedicated group, while the remaining six themes were paired across three discussion groups ((a) + (b); (f) + (g); (h) + (i)). Each group collaboratively shortlisted up to five critical questions within their allocated theme(s), judged on importance, novelty, feasibility and relevance.

The resulting shortlist of 25 questions (Supplementary Table 2) was ranked anonymously using the Mentimeter interactive response system (www.mentimeter.com). Participants independently assigned priority scores to each question, ranging from 1 (lowest priority) to 100 (highest priority), drawing on their professional judgement of each question’s importance and potential to advance BCE science and policy. Mean scores were used to identify the top ten highest-ranked questions, with results revealed only after voting closed to minimize bias.

A structured plenary session was held to refine the wording of the top-ten-ranked questions, without altering their original rank order (see Supplementary Text 3 for review procedure). Where relevant, overlapping elements from lower-ranked questions were integrated into related higher-ranked ones to improve coherence and breadth. No formal roles (for example, cynics versus advocates) were assigned, and discussion focused on phrasing refinement, rather than consensus-seeking.

### Characterization of priority questions

To strengthen the relevance and applicability of the final outputs, a subsequent expert assessment evaluated the top ten research questions across four practical dimensions: (1) timescale, (2) research complexity, (3) cost and (4) policy relevance. Participants independently scored each dimension using a predefined three-tier system (low, medium and high; see Table 1 for definitions of each dimension and tier). Modal scores for each question–dimension pair were used as the representative score, with ties retained where no single mode emerged. See Supplementary Data 1 for scoring distributions.

The manuscript was developed through collaborative writing, with experts working in teams to draft pre-assigned sections based on their expertise and interest. All participants that contributed to the identification, ranking and manuscript preparation are listed as co-authors. The top ten questions are referenced in the text by rank order (for example, Q1, Q2), with wording refined during the review process. Although often interconnected, each question addresses a distinct challenge and collectively builds a coherent roadmap for advancing blue carbon science and policy.

### Reporting summary

Further information on research design is available in the Nature Portfolio Reporting Summary linked to this article.

## Supplementary information

Supplementary Information Supplementary Text 1–3 and Tables 1 and 2.

Reporting Summary

Supplementary Data 1 Excel file comprising: (Tab 1) the full set of submitted research questions with thematic categorization; (Tab 2) participant worksheets used for scoring questions across evaluation dimensions; and (Tab 3) aggregated results of the dimension scoring.

## Data Availability

All data generated for this study are available in Supplementary Data 1.

## References

[1] Nellemann, C. et al. *Blue Carbon: The Role of Healthy Oceans in Binding Carbon - A Rapid Response Assessment* (United Nations Environment Programme, GRID-Arendal, 2009).

[2] Macreadie, P. I. et al. Blue carbon as a natural climate solution. *Nat. Rev. Earth Env.* **2**, 826–839 (2021).

[3] Duarte, C. M., Losada, I. J., Hendriks, I. E., Mazarrasa, I. & Marbà, N. The role of coastal plant communities for climate change mitigation and adaptation. *Nat. Clim. Change* **3**, 961–968 (2013).

[4] James, K., Macreadie, P. I., Burdett, H. L., Davies, I. & Kamenos, N. A. It’s time to broaden what we consider a ‘blue carbon ecosystem’. *Glob. Change Biol.*10.1111/gcb.17261 (2024). 10.1111/gcb.17261 38712641

[5] Adame, M. F. et al. All tidal wetlands are blue carbon ecosystems. *Bioscience* **74**, 253–268 (2024). 38720908 10.1093/biosci/biae007 PMC11075650

[6] IPCC*. 2013 Supplement to the**2006 IPCC Guidelines for National Greenhouse Gas Inventories: Wetlands* (IPCC, 2014).

[7] Kelleway, J. J. et al. A national approach to greenhouse gas abatement through blue carbon management. *Glob. Environ. Change* **63**, 102083 (2020).

[8] Lovelock, C. E. et al. An Australian blue carbon method to estimate climate change mitigation benefits of coastal wetland restoration. *Restor. Ecol.* **31**, e13739 (2023).

[9] Herr, D. & Landis, E. *Coastal Blue Carbon Ecosystems Opportunities for Nationally Determined Contributions* (IUCN & The Nature Conservancy, 2016).

[10] *International Policy Framework for Blue Carbon Ecosystems: Recommendations to Align Actions across International Policy Processes for the Conservation and Restoration of Coastal Blue Carbon Ecosystems* (IUCN & Conservation International, 2023).

[11] Macreadie, P. I. et al. The future of Blue Carbon science. *Nat. Commun.* **10**, 3998 (2019). 31488846 10.1038/s41467-019-11693-w PMC6728345

[12] Costa, M. D. P. & Macreadie, P. I. The evolution of Blue Carbon science. *Wetlands* **42**, 109 (2022).

[13] Al-Haj, A. N. & Fulweiler, R. W. A synthesis of methane emissions from shallow vegetated coastal ecosystems. *Glob. Change Biol.* **26**, 2988–3005 (2020). 10.1111/gcb.15046 32068924

[14] Kennedy, H. et al. Species traits and geomorphic setting as drivers of global soil carbon stocks in seagrass meadows. *Global Biogeochem. Cycles*10.1029/2022GB007481 (2022).

[15] Maxwell, T. L. et al. Soil carbon in the world’s tidal marshes. *Nat. Commun.* **15**, 10265 (2024). 39592604 10.1038/s41467-024-54572-9 PMC11599748

[16] Bunting, P. et al. Global mangrove extent change 1996–2020: Global Mangrove Watch version 3.0. *Remote Sens.* **14**, 3657 (2022).

[17] Dunic, J. C., Brown, C. J., Connolly, R. M., Turschwell, M. P. & Côté, I. M. Long-term declines and recovery of meadow area across the world’s seagrass bioregions. *Glob. Change Biol.* **27**, 4096–4109 (2021). 10.1111/gcb.15684 33993580

[18] Costa, M. D. P. et al. Modelling blue carbon farming opportunities at different spatial scales. *J. Environ. Manage.* **301**, 113813 (2022). 34607133 10.1016/j.jenvman.2021.113813

[19] Moritsch, M. M. et al. Estimating blue carbon sequestration under coastal management scenarios. *Sci. Total Environ.* **777**, 145962 (2021). 33684760 10.1016/j.scitotenv.2021.145962

[20] Williamson, P. & Gattuso, J. P. Carbon removal using coastal blue carbon ecosystems is uncertain and unreliable, with questionable climatic cost-effectiveness. *Front. Clim.* **4**, 853666 (2022).

[21] Johannessen, S. C. & Christian, J. R. Why blue carbon cannot truly offset fossil fuel emissions. *Commun. Earth Environ.*10.1038/s43247-023-01068-x (2023).

[22] Kristensen, E., Flindt, M. R. & Quintana, C. O. Predicting climate mitigation through carbon burial in blue carbon ecosystems—challenges and pitfalls. *Glob. Change Biol.* **31**, e70022 (2025). 10.1111/gcb.70022 39757865

[23] Fu, C. C., Steckbauer, A., Mann, H. & Duarte, C. M. Achieving the Kunming-Montreal global biodiversity targets for blue carbon ecosystems. *Nat. Rev. Earth Env.* **5**, 538–552 (2024).

[24] IPCC. *Special Report on the Ocean and Cryosphere in a Changing Climate* (eds Pörtner, H.O. et al.) (Cambridge Univ. Press, 2019).

[25] Friess, D. The potential for mangrove and seagrass blue carbon in Small Island States. *Curr. Opin. Environ. Sustain.*10.1016/j.cosust.2023.101324 (2023).

[26] Sutherland, W. J., Fleishman, E., Mascia, M. B., Pretty, J. & Rudd, M. A. Methods for collaboratively identifying research priorities and emerging issues in science and policy. *Methods Ecol. Evol.* **2**, 238–247 (2011).

[27] Seddon, A. W. R. et al. Looking forward through the past: identification of 50 priority research questions in palaeoecology. *J. Ecol.* **102**, 256–267 (2014).

[28] Vierros, M. Communities and blue carbon: the role of traditional management systems in providing benefits for carbon storage, biodiversity conservation and livelihoods. *Clim. Change* **140**, 89–100 (2017).

[29] Albuquerque, U. P. et al. Integrating traditional ecological knowledge into academic research at local and global scales. *Reg. Environ. Change* **21**, 1–11 (2021).33362432

[30] Govan, H. et al. *Status and Potential of Locally-Managed Marine Areas in the South Pacific: Meeting Nature Conservation and Sustainable Livelihood Targets Through Wide-Spread Implementation of LMMAs: Study Report* (SPREP/WWF/WorldFish-Reefbase/CRISP, 2009).

[31] O’Garra, T. et al. National-level evaluation of a community-based marine management initiative. *Nat. Sustain.* **6**, 908–918 (2023).

[32] Grimm, K. et al. *Including Local Ecological Knowledge (LEK) in Mangrove Conservation & Restoration. A Best-Practice Guide for Practitioners and Researchers* (Global Mangrove Alliance, 2024).

[33] Wylie, L., Sutton-Grier, A. E. & Moore, A. Keys to successful blue carbon projects: lessons learned from global case studies. *Mar. Policy* **65**, 76–84 (2016).

[34] Cisneros-Montemayor, A. M. et al. Agreements and benefits in emerging ocean sectors: are we moving towards an equitable Blue Economy?. *Ocean Coast. Manag.*10.1016/j.ocecoaman.2022.106097 (2022).

[35] Conservation International et al. *High-Quality Blue Carbon Principles and Guidance* (Ocean Risk and Resilience Action Alliance, 2022).

[36] Cisneros-Montemayor, A. M. et al. Enabling conditions for an equitable and sustainable blue economy. *Nature* **591**, 396–401 (2021). 33731948 10.1038/s41586-021-03327-3

[37] van Katwijk, M. M. et al. Global analysis of seagrass restoration: the importance of large-scale planting. *J. Appl. Ecol.* **53**, 567–578 (2016).

[38] Hemraj, D. A. et al. Nature protection must precede restoration. *Science* **383**, 158 (2024). 38207034 10.1126/science.adn0543

[39] Bayraktarov, E. et al. The cost and feasibility of marine coastal restoration. *Ecol. Appl.* **26**, 1055–1074 (2016). 27509748 10.1890/15-1077

[40] Gatt, Y. M. et al. The Mangrove Restoration Tracker Tool: meeting local practitioner needs and tracking progress toward global targets. *One Earth* **7**, 2072–2085 (2024).

[41] Lee, S. Y., Hamilton, S., Barbier, E. B., Primavera, J. & Lewis, R. R. Better restoration policies are needed to conserve mangrove ecosystems. *Nat. Ecol. Evol.* **3**, 870–872 (2019). 31036899 10.1038/s41559-019-0861-y

[42] Saunders, M. I. et al. Bright spots in coastal marine ecosystem restoration. *Curr. Biol.* **30**, R1500–R1510 (2020). 33352137 10.1016/j.cub.2020.10.056

[43] Bourgeois, C. F. et al. Four decades of data indicate that planted mangroves stored up to 75% of the carbon stocks found in intact mature stands. *Sci. Adv.* **10**, eadk5430 (2024). 38968357 10.1126/sciadv.adk5430 PMC11801255

[44] Lewis Iii, R. R. Ecological engineering for successful management and restoration of mangrove forests. *Ecol. Eng.* **24**, 403–418 (2005).

[45] Brown, B., Fadillah, R., Nurdin, Y., Soulsby, I. & Ahmad, R. Case study: Community Based Ecological Mangrove Rehabilitation (CBEMR) in Indonesia. From small (12–33 ha) to medium scales (400 ha) with pathways for adoption at larger scales (> 5000 ha). *S.A.P.I.EN.S*. http://journals.openedition.org/sapiens/1589 (2014).

[46] Duarte, C. M., Apostolaki, E. T., Serrano, O., Steckbauer, A. & Unsworth, R. K. Conserving seagrass ecosystems to meet global biodiversity and climate goals. *Nat. Rev. Biodivers.*10.1038/s44358-025-00028-x (2025).

[47] Flindt, M. et al. *Human Impacts, Environmental Disturbances, and Restoration of Seagrasses* (Elsevier, 2024).

[48] Alongi, D. M. Nitrogen cycling and mass balance in the world’s mangrove forests. *Nitrogen* **1**, 167–189 (2020).

[49] Helton, A. M., Ardón, M. & Bernhardt, E. S. Thermodynamic constraints on the utility of ecological stoichiometry for explaining global biogeochemical patterns. *Ecol. Lett.* **18**, 1049–1056 (2015). 26259672 10.1111/ele.12487

[50] Fonseca, M. S., Kenworthy, W. J., Courtney, F. X. & Hall, M. O. Seagrass planting in the southeastern United States: methods for accelerating habitat development. *Restor. Ecol.* **2**, 198–212 (1994).

[51] Piazzi, L. et al. Environmental engineering techniques to restore degraded *Posidonia oceanica* meadows. *Water* **13**, 661 (2021).

[52] Pétillon, J. et al. Top ten priorities for global saltmarsh restoration, conservation and ecosystem service research. *Sci. Total Environ.* **898**, 165544 (2023). 37453706 10.1016/j.scitotenv.2023.165544

[53] Wang, J.-J., Li, X.-Z., Lin, S.-W. & Ma, Y.-X. Economic evaluation and systematic review of salt marsh restoration projects at a global scale. *Front. Ecol. Evol.* **10**, 865516 (2022).

[54] Callaway, J. C., Borgnis, E. L., Turner, R. E. & Milan, C. S. Carbon sequestration and sediment accretion in San Francisco Bay tidal wetlands. *Estuaries Coasts* **35**, 1163–1181 (2012).

[55] Crameri, N. J. et al. Feral ungulate impacts on carbon cycling in a coastal floodplain wetland in tropical northern Australia. *J. Geophys. Res.: Biogeosciences* **130**, e2025JG009056 (2025).

[56] Balaji, V., Sekar, V. & Murugesan, G. Comparison of seagrass restoration methods adopted in Palk Bay, India. *J. Mar. Biol. Assoc. India* **62**, 96 (2020).

[57] Kairo, J. G. & Mangora, M. M. *Guidelines on Mangrove Ecosystem Restoration for the Western Indian Ocean Region – Western Indian Ocean Ecosystem Guidelines and Toolkits* (United Nations Environment Programme/Nairobi Convention Secretariat, 2020): https://www.nairobiconvention.org/CHM%20Documents/WIOSAP/guidelines/GuidelinesonMangroveRestorationForTheWIO.pdf

[58] Spivak, A. C., Sanderman, J., Bowen, J. L., Canuel, E. A. & Hopkinson, C. S. Global-change controls on soil-carbon accumulation and loss in coastal vegetated ecosystems. *Nat. Geosci.* **12**, 685–692 (2019).

[59] Lovelock, C. E. & Reef, R. Variable impacts of climate change on blue carbon. *One Earth* **3**, 195–211 (2020).

[60] Kirwan, M. L., Megonigal, J. P., Noyce, G. L. & Smith, A. J. Geomorphic and ecological constraints on the coastal carbon sink. *Nat. Rev. Earth Environ.* **4**, 393–406 (2023).

[61] Gu, J. L. & Wu, J. P. Blue carbon effects of mangrove restoration in subtropics where Spartina alterniflora invaded. *Ecol. Eng.*10.1016/j.ecoleng.2022.106822 (2023).

[62] Canal-Vergés, P. et al. Impacts of sea level rise on Danish coastal wetlands–a GIS-based analysis. *Environ. Manag.* **75**, 1039–1054 (2025). 10.1007/s00267-024-02096-9 PMC11965245 39611951

[63] Schuerch, M. et al. Large-scale loss of Mediterranean coastal marshes under rising sea levels by 2100. *Commun. Earth Environ.* **6**, 128 (2025).

[64] Young, M. A. et al. National scale predictions of contemporary and future blue carbon storage. *Sci. Total Environ.* **800**, 149573 (2021). 34399348 10.1016/j.scitotenv.2021.149573

[65] Verified Carbon Standard: Program Guide v.4.4 (VERRA, 2023).

[66] Chen, R. & Twilley, R. R. A simulation model of organic matter and nutrient accumulation in mangrove wetland soils. *Biogeochemistry* **44**, 93–118 (1999).

[67] Lee, H. et al. *Sea Level Affecting Marshes Model (SLAMM) – New Functionality for Predicting Changes in Distribution of Submerged Aquatic Vegetation in Response to Sea Level Rise. Version 1.0.* (US Environmental Protection Agency, 2014).

[68] Swanson, K. M. et al. Wetland accretion rate model of ecosystem resilience (WARMER) and its application to habitat sustainability for endangered species in the San Francisco estuary. *Estuaries Coasts* **37**, 476–492 (2014).

[69] Vahsen, M. L. et al. Cohort Marsh Equilibrium Model (CMEM): history, mathematics, and implementation. *J. Geophys. Res.: Biogeosciences* **129**, e2023JG007823 (2024).

[70] Morris, J. T. & Sundberg, K. Responses of coastal wetlands to rising sea-level revisited: the importance of organic production. *Estuaries Coast.* **47**, 1735–1749 (2024).

[71] Malerba, M. E. et al. Methane and nitrous oxide emissions complicate the climate benefits of teal and blue carbon wetlands. *One Earth* **5**, 1336–1341 (2022).

[72] Reithmaier, G. M. S. et al. Carbonate chemistry and carbon sequestration driven by inorganic carbon outwelling from mangroves and saltmarshes. *Nat. Commun.*10.1038/s41467-023-44037-w (2023). 10.1038/s41467-023-44037-w PMC10713528 38081846

[73] Oikawa, P. Y. et al. A new coupled biogeochemical modeling approach provides accurate predictions of methane and carbon dioxide fluxes across diverse tidal wetlands. *J. Geophys. Res.: Biogeosciences*10.1029/2023JG007943 (2024).

[74] Dai, Z. H., Trettin, C. C., Frolking, S. & Birdsey, R. A. Mangrove carbon assessment tool: model validation and assessment of mangroves in southern USA and Mexico. *Estuar. Coast Shelf Sci.* **208**, 107–117 (2018).

[75] Tang, F. et al. Modelling long-term soil organic carbon dynamics of the typical salt marsh wetlands with the DNDC model: a case study from Yancheng wetland, China. *Ecol. Model.* **510**, 111288 (2025).

[76] Wang, H. et al. Modeling impacts of drought-induced salinity intrusion on carbon dynamics in tidal freshwater forested wetlands. *Ecol. Appl.* **32**, e2700 (2022). 35751513 10.1002/eap.2700

[77] Bunsen, F., Nissen, C. & Hauck, J. The impact of recent climate change on the global ocean carbon sink. *Geophys. Res. Lett.* **51**, e2023GL107030 (2024).

[78] Tan, L. et al. Conversion of coastal wetlands, riparian wetlands, and peatlands increases greenhouse gas emissions: a global meta-analysis. *Glob. Change Biol.* **26**, 1638–1653 (2020). 10.1111/gcb.14933 31755630

[79] Holmquist, J. R. et al. The Coastal Carbon Library and Atlas: open source soil data and tools supporting blue carbon research and policy. *Glob. Change Biol.*10.1111/gcb.17098 (2024). 10.1111/gcb.17098 38273507

[80] Maxwell, T. L. et al. Global dataset of soil organic carbon in tidal marshes. *Sci. Data*10.1038/s41597-023-02633-x (2023). 10.1038/s41597-023-02633-x PMC10640612 37952023

[81] Rovai, A. S. et al. Global controls on carbon storage in mangrove soils. *Nat. Clim. Change* **8**, 534–538 (2018).

[82] Stankovic, M. et al. Blue carbon assessments of seagrass and mangrove ecosystems in South and Southeast Asia: current progress and knowledge gaps. *Sci. Total Environ.*10.1016/j.scitotenv.2023.166618 (2023). 10.1016/j.scitotenv.2023.166618 37643707

[83] Hatje, V. et al. Vegetated coastal ecosystems in the Southwestern Atlantic Ocean are an unexploited opportunity for climate change mitigation. *Commun. Earth Environ.*10.1038/s43247-023-00828-z (2023).

[84] Holmquist, J. R. et al. Uncertainty in United States coastal wetland greenhouse gas inventorying. *Environ. Res. Lett.* **13**, 115005 (2018).

[85] Arias-Ortiz, A. et al. Methane fluxes in tidal marshes of the conterminous United States. *Glob. Change Biol.*10.1111/gcb.17462 (2024). 10.1111/gcb.17462 39234688

[86] Rosentreter, J. A. et al. Coastal vegetation and estuaries are collectively a greenhouse gas sink. *Nat. Clim. Change* **13**, 579–587 (2023).

[87] Kirwan, M. L. & Megonigal, J. P. Tidal wetland stability in the face of human impacts and sea-level rise. *Nature* **504**, 53–60 (2013). 24305148 10.1038/nature12856

[88] Rogers, K. et al. Wetland carbon storage controlled by millennial-scale variation in relative sea-level rise. *Nature* **567**, 91–95 (2019). 30842636 10.1038/s41586-019-0951-7

[89] Simpson, L. T., Osborne, T. Z. & Feller, I. C. Wetland soil Co_2_ efflux along a latitudinal gradient of spatial and temporal complexity. *Estuaries Coasts* **42**, 45–54 (2019).

[90] Morris, J. T., Sundareshwar, P. V., Nietch, C. T., Kjerfve, B. & Cahoon, D. R. Responses of coastal wetlands to rising sea level. *Ecology* **83**, 2869–2877 (2002).

[91] Kirwan, M. L. & Mudd, S. M. Response of salt-marsh carbon accumulation to climate change. *Nature* **489**, 550 (2012). 23018965 10.1038/nature11440

[92] Rietl, A. J., Megonigal, J. P., Herbert, E. R. & Kirwan, M. L. Vegetation type and decomposition priming mediate brackish marsh carbon accumulation under interacting facets of global change. *Geophys. Res. Lett.* **48**, e2020GL092051 (2021).

[93] Derby, R. K., Needelman, B. A., Roden, A. A. & Megonigal, J. P. Vegetation and hydrology stratification as proxies to estimate methane emission from tidal marshes. *Biogeochemistry* **157**, 227–243 (2022).

[94] Koontz, E. L. et al. Controls on spatial variation in porewater methane concentrations across United States tidal wetlands. *Sci. Total Environ.* **957**, 177290 (2024). 39491559 10.1016/j.scitotenv.2024.177290

[95] Ollivier, Q. R., Maher, D. T., Pitfield, C. & Macreadie, P. Net drawdown of greenhouse gases (CO_2_, CH_4_ and N_2_O) by a temperate Australian seagrass meadow. *Estuaries Coasts* **45**, 2026–2039 (2022).

[96] Moseman-Valtierra, S. et al. Substantial nitrous oxide emissions from intertidal sediments and groundwater in anthropogenically-impacted West Falmouth Harbor, Massachusetts. *Chemosphere* **119**, 1281–1288 (2015). 25460773 10.1016/j.chemosphere.2014.10.027

[97] Malerba, M. E. et al. Conserving nature’s chorus: local and landscape features promoting frog species richness in farm dams. *Biol. Conserv.*10.1016/j.biocon.2023.110270 (2023).

[98] Wilkinson, M. D. et al. The FAIR Guiding Principles for scientific data management and stewardship. *Sci. Data* **3**, 1–9 (2016). 10.1038/sdata.2016.18 PMC4792175 26978244

[99] United Nations, European Commission, Food and Agricultural Organization of the United Nations, Organisation for Economic Co-operation and Development, United Nations Environment Programme, and World Bank. *System of Environmental-Economic Accounting – Ecosystem Accounting (SEEA EA)*10.5089/9789212591834.069 (UN, 2021).

[100] *An Introduction to Ecosystem Accounting: Key Concepts and Policy Applications* (United Nations, 2021).

[101] Lammerant, J. State of play of business accounting and reporting on ecosystems. In *Forum of Experts in SEEA Experimental Ecosystem Accounting* (UN, 2019).

[102] Bertram, C. et al. The blue carbon wealth of nations. *Nat. Clim. Change* **11**, 704 (2021).

[103] Hernández-Blanco, M., Costanza, R. & Cifuentes-Jara, M. Economic valuation of the ecosystem services provided by the mangroves of the Gulf of Nicoya using a hybrid methodology. *Ecosyst. Serv.* **49**, 101258 (2021).

[104] Carnell, P. et al. *Measuring and Accounting for the Benefits of Restoring Blue Carbon Ecosystems: The Guide* (Department of Climate Change, Energy, the Environment and Water, Canberra, Australia, 2024).

[105] Glamore, W. et al. *Accounting for the Benefits from Coastal Restoration: A Case Study from the Hunter River* (Department of Climate Change, Energy, the Environment and Water, Canberra, Australia, 2024).

[106] Nursey-Bray, M. et al. *Accounting for the Benefits from Coastal restoration: A Case Study from East Trinity Inlet* (Department of Climate Change, Energy, the Environment and Water, Canberra, Australia, 2024).

[107] Lovelock, C. E. & Duarte, C. M. Dimensions of Blue Carbon and emerging perspectives. *Biol. Lett.*10.1098/rsbl.2018.0781 (2019). 10.1098/rsbl.2018.0781 PMC6451379 30836882

[108] Friess, D. A., Howard, J., Huxham, M., Macreadie, P. I. & Ross, F. Capitalizing on the global financial interest in blue carbon. *PLoS Clim.* **1**, e0000061 (2022).

[109] Araya-Lopez, R., Costa, M. D. D., Wartman, M. & Macreadie, P. I. Trends in the application of remote sensing in blue carbon science. *Ecol. Evol.*10.1002/ece3.10559 (2023). 10.1002/ece3.10559 PMC10517596 37745789

[110] Pham, T. D. et al. Advances in Earth observation and machine learning for quantifying blue carbon. *Earth-Sci. Rev.*10.1016/j.earscirev.2023.104501 (2023).

[111] Bansal, S. et al. Practical guide to measuring wetland carbon pools and fluxes. *Wetlands* **43**, 105 (2023). 38037553 10.1007/s13157-023-01722-2 PMC10684704

[112] Mayen, J. et al. Atmospheric CO_2_ exchanges measured by eddy covariance over a temperate salt marsh and influence of environmental controlling factors. *Biogeosciences* **21**, 993–1016 (2024).

[113] Adame, M. F. et al. Deconstructing the mangrove carbon cycle: gains, transformation, and losses. *Ecosphere*10.1002/ecs2.4806 (2024).

[114] Dahl, M. et al. Recommendations for strengthening blue carbon science. *One Earth* **8** (2025).

[115] Howard, J., Hoyt, S., Isensee, K., Telszewski, M. & Pidgeon, E. *Coastal Blue Carbon: Methods for Assessing Carbon Stocks and Emissions Factors in Mangroves, Tidal Salt Marshes, and Seagrasses* (Conservation International, Intergovernmental Oceanographic Commission of UNESCO, International Union for Conservation of Nature, 2014).

[116] Eyre, B. D., Camillini, N., Glud, R. N. & Rosentreter, J. A. The climate benefit of seagrass blue carbon is reduced by methane fluxes and enhanced by nitrous oxide fluxes. *Commun. Earth Environ.*10.1038/s43247-023-01022-x (2023).

[117] Malerba, M. E. et al. Remote sensing for cost-effective blue carbon accounting. *Earth-Sci. Rev.*10.1016/j.earscirev.2023.104337 (2023).

[118] Pham, D. T. et al. A review of remote sensing approaches for monitoring blue carbon ecosystems: mangroves, seagrasses and salt marshes during 2010–2018. *Sensors*10.3390/s19081933 (2019). 10.3390/s19081933 PMC6515341 31022958

[119] Campbell, A. D., Fatoyinbo, L., Goldberg, L. & Lagomasino, D. Global hotspots of salt marsh change and carbon emissions. *Nature* **612**, 701 (2022). 36450979 10.1038/s41586-022-05355-z PMC9771810

[120] Richards, D. R., Thompson, B. S. & Wijedasa, L. Quantifying net loss of global mangrove carbon stocks from 20 years of land cover change. *Nat. Commun.* **11**, 4260 (2020). 32848150 10.1038/s41467-020-18118-z PMC7450071

[121] Perera, N. S., Costa, M. D. P., Macreadie, P. I. & Wartman, M. Trends in market-based blue carbon projects. *Sustain. Dev.*10.1002/sd.3293 (2024).

[122] Atwood, T. B. et al. Global patterns in mangrove soil carbon stocks and losses. *Nat. Clim. Change* **7**, 523 (2017).

[123] Hamilton, S. E. & Friess, D. A. Global carbon stocks and potential emissions due to mangrove deforestation from 2000 to 2012. *Nat. Clim. Change* **8**, 240 (2018).

[124] Sanderman, J. et al. A global map of mangrove forest soil carbon at 30 m spatial resolution. *Environ. Res. Lett.*10.1088/1748-9326/aabe1c (2018).

[125] Houston, A., Garnett, M. H. & Austin, W. E. N. Blue carbon additionality: new insights from the radiocarbon content of saltmarsh soils and their respired CO_2_. *Limnol. Oceanogr.* **69**, 548–561 (2024).

[126] Graversen, A. E. L. et al. A marine and salt marsh sediment organic carbon database for European regional seas (EURO-CARBON). *Data Br.* **60**, 111595 (2025). 10.1016/j.dib.2025.111595 PMC12149566 40496737

[127] Wolfe, J. & Holmquist, J. *Supplemental Figures for ‘Recharting Blue Carbon Science: Priority Questions for the Next Decade’* (Smithsonian Environmental Research Center, 2025).

[128] Veettil, B. K. et al. Blue carbon ecosystems in Sri Lanka: a review. *Estuar. Coast Shelf Sci.*10.1016/j.ecss.2024.108907 (2024).

[129] Oreska, M. P. J. et al. The greenhouse gas offset potential from seagrass restoration. *Sci. Rep.* **10**, 7325 (2020). 32355280 10.1038/s41598-020-64094-1 PMC7193639

[130] Bogard, M. J. et al. Hydrologic export is a major component of coastal wetland carbon budgets. *Glob. Biogeochem. Cycles* **34**, e2019GB006430 (2020).

[131] Piñeiro-Juncal, N. et al. Soil organic carbon depth profiles and centennial and millennial decay rates in tidal marsh, mangrove and seagrass blue carbon ecosystems. *Commun. Earth Environ.* **6**, 504 (2025).

[132] Krauss, K. W. et al. The role of the upper tidal estuary in wetland blue carbon storage and flux. *Glob. Biogeochem. Cycles* **32**, 817–839 (2018).

[133] Dahl, M. et al. First assessment of seagrass carbon accumulation rates in Sweden: a field study from a fjord system at the Skagerrak coast. *PLoS Clim.* **2**, e0000099 (2023).

[134] Krause-Jensen, D. et al. Nordic blue carbon ecosystems: status and outlook. *Front. Mar. Sci.*10.3389/fmars.2022.847544 (2022).

[135] Leiva-Dueñas, C. et al. Region-specific drivers cause low organic carbon stocks and sequestration rates in the saltmarsh soils of southern Scandinavia. *Limnol. Oceanogr.* **69**, 290–308 (2024).

[136] Worthington, T. A. et al. The distribution of global tidal marshes from earth observation data. *Glob. Ecol. Biogeogr.* **33**, e13852 (2024).

[137] Twilley, R. R., Rovai, A. S. & Riul, P. Coastal morphology explains global blue carbon distributions. *Front. Ecol. Environ.* **16**, 503–508 (2018).

[138] Worthington, T. A. et al. A global biophysical typology of mangroves and its relevance for ecosystem structure and deforestation. *Sci. Rep.* **10**, 14652 (2020). 32887898 10.1038/s41598-020-71194-5 PMC7473852

[139] Breithaupt, J. L. & Steinmuller, H. E. Refining the global estimate of mangrove carbon burial rates using sedimentary and geomorphic settings. *Geophys. Res. Lett.*10.1029/2022GL100177 (2022).

[140] Ouyang, X. & Lee, S. Y. Improved estimates on global carbon stock and carbon pools in tidal wetlands. *Nat. Commun.* **11**, 317 (2020). 31949151 10.1038/s41467-019-14120-2 PMC6965625

[141] Santos, R. et al. Superficial sedimentary stocks and sources of carbon and nitrogen in coastal vegetated assemblages along a flow gradient. *Sci. Rep.*10.1038/s41598-018-37031-6 (2019). 10.1038/s41598-018-37031-6 PMC6345834 30679706

[142] Pessarrodona, A. et al. Global seaweed productivity. *Sci. Adv.*10.1126/sciadv.abn2465 (2022). 10.1126/sciadv.abn2465 PMC9473579 36103524

[143] McKenzie, L. J. et al. The global distribution of seagrass meadows. *Environ. Res. Lett.* **15**, 074041 (2020).

[144] Kearney, M. & Porter, W. Mechanistic niche modelling: combining physiological and spatial data to predict species’ ranges. *Ecol. Lett.* **12**, 334–350 (2009). 19292794 10.1111/j.1461-0248.2008.01277.x

[145] Bertelli, C. M., Stokes, H. J., Bull, J. C. & Unsworth, R. K. F. The use of habitat suitability modelling for seagrass: a review. *Front. Mar. Sci.* **9** (2022).

[146] Jayathilake, D. R. M. & Costello, M. J. A modelled global distribution of the kelp biome. *Biol. Conserv.* **252**, 108815 (2020).

[147] Duarte, C. M. et al. Global estimates of the extent and production of macroalgal forests. *Glob. Ecol. Biogeogr.* **31**, 1422–1439 (2022).

[148] Rodríguez-Medina, K., Yañez-Arenas, C., Peterson, A. T., Euán Ávila, J. & Herrera-Silveira, J. Evaluating the capacity of species distribution modeling to predict the geographic distribution of the mangrove community in Mexico. *PLoS ONE* **15**, e0237701 (2020). 32817628 10.1371/journal.pone.0237701 PMC7446832

[149] Palacios, M. F., Baumgartner, K. L., Laidre, K. L. & Gregr, E. J. Beyond correlation: integrating environmentally and behaviourally mediated processes in models of marine mammal distributions. *Endanger. Species Res.* **22**, 191–203 (2013).

[150] Strong-Wright, J. & Taylor, J. R. Modeling the growth potential of the kelp *Saccharina latissima* in the North Atlantic. *Front. Mar. Sci.* **8** (2022).

[151] Wilson, K. L., Wong, M. C. & Devred, E. *Mapping Eelgrass Habitat in Southern Nova Scotia with Worldview-2/3 Satellite Imagery* (Fisheries and Oceans Canada, 2024).

[152] Queirós, A. M. et al. Connected macroalgal-sediment systems: blue carbon and food webs in the deep coastal ocean. *Ecol. Monogr.*10.1002/ecm.1366 (2019).

[153] Filbee-Dexter, K. et al. Carbon export from seaweed forests to deep ocean sinks. *Nat. Geosci.*10.1038/s41561-024-01449-7 (2024).

[154] Aldy, J. E., Krupnick, A. J., Newell, R. G., Parry, I. W. H. & Pizer, W. A. Designing climate mitigation policy. *J. Econ. Lit.* **48**, 903–934 (2010).

[155] Howard, J. et al. Blue carbon pathways for climate mitigation: known, emerging and unlikely. *Mar. Policy*10.1016/j.marpol.2023.105788 (2023).

[156] Himes-Cornell, A., Grose, S. O. & Pendleton, L. Mangrove ecosystem service values and methodological approaches to valuation: where do we stand? *Front. Mar. Sci.*10.3389/fmars.2018.00376 (2018).

[157] Hagger, V., Waltham, N. J. & Lovelock, C. E. Opportunities for coastal wetland restoration for blue carbon with co-benefits for biodiversity, coastal fisheries, and water quality. *Ecosyst. Serv.*10.1016/j.ecoser.2022.101423 (2022).

[158] Hejnowicz, A. P., Kennedy, H., Rudd, M. A. & Huxham, M. R. Harnessing the climate mitigation, conservation and poverty alleviation potential of seagrasses: prospects for developing blue carbon initiatives and payment for ecosystem service programmes. *Front. Mar. Sci.* **2**, 1–22 (2015).

[159] Christianson, A. B. et al. The promise of blue carbon climate solutions: where the science supports ocean-climate policy. *Front. Mar. Sci.*10.3389/fmars.2022.851448 (2022).

[160] Regnier, P., Resplandy, L., Najjar, R. G. & Ciais, P. The land-to-ocean loops of the global carbon cycle. *Nature* **603**, 401–410 (2022). 35296840 10.1038/s41586-021-04339-9

[161] Giri, C. et al. Status and distribution of mangrove forests of the world using earth observation satellite data. *Glob. Ecol. Biogeogr.* **20**, 154–159 (2011).

[162] Bunting, P. et al. The Global Mangrove Watch—a new 2010 global baseline of mangrove extent. *Remote Sens.* **10** (2018).

[163] Murray, N. J. et al. The global distribution and trajectory of tidal flats. *Nature* **565**, 222–225 (2019). 30568300 10.1038/s41586-018-0805-8

[164] Murray, N. J. et al. High-resolution global maps of tidal flat ecosystems from 1984 to 2019. *Sci. Data* **9**, 542 (2022). 36068234 10.1038/s41597-022-01635-5 PMC9448797

[165] McOwen, C. J. et al. A global map of saltmarshes. *Biodivers. Data J.*10.3897/BDJ.5.e11764 (2017). 10.3897/BDJ.5.e11764 PMC5515097 28765720

[166] Arias-Ortiz, A. et al. Seagrass sediment organic carbon burial rates are globally significant. Preprint at *Research Square*10.21203/rs.3.rs-8462059/v1 (2026).

[167] Xiong, Y. et al. Global patterns of tree stem growth and stand aboveground wood production in mangrove forests. *For. Ecol. Manag.* **444**, 382–392 (2019).

