# Supplementary material for: Priority questions for the next decade of blue carbon science
Source: Nat Ecol Evol. 2026 Mar 24;10(4):751–64. doi: 10.1038/s41559-026-03020-6 (PMC13076200; doi:10.1038/s41559-026-03020-6)
Supplement: Supplementary file 1 — Supplementary Text 1–3 and Tables 1 and 2. [file 41559_2026_3020_MOESM1_ESM.pdf]

nature ecology & evolution

Supplementary information https://doi.org/10.1038/s41559-026-03020-6

Priority questions for the next decade of blue carbon science

In the format provided by the authors and unedited

SUPPLEMENTARY INFORMATION

Supplementary Text S1. Expert Background

Participants spanned six global regions, including Australasia (Australia), North and Central America (United States, Costa Rica, Panama, Mexico), South America (Brazil), Europe (United Kingdom, Spain, Denmark, Monaco, France), Africa (South Africa, Tanzania), and Asia (Japan, China, Thailand).

Supplementary Text S2. Thematic shortlisting of priority questions

The 116 questions submitted were consolidated to remove any duplicates and grouped into nine thematic categories to facilitate discussion and prioritisation (See Supplementary Data). Themes included: (A) Boundaries & Definitions: Clarifying scope and terminology of blue carbon; (B) Emerging BCEs: Including non-classical, emerging BCEs; (C) Prediction: Modelling variability and climate change, forecasting, scaling, and drivers; (D) Measurement: Inventories, mapping, monitoring, and data management; (E) Crediting & Standards: Carbon credit rules and MRV requirements; (F) Co-Benefits: Non-carbon services like biodiversity, coastal protection, and nutrient cycling; (G) Communication: Conduct, awareness, transparent messaging, managing expectations; (H) Finance & Markets: Investment, project affordability, feasibility, and financing mechanism; (I) Social & Policy: Communities, equity, policy frameworks, global agendas, and governance.

Participants were then assigned to six discussion groups (4-6 experts each) structured to ensure diversity of expertise, geographic representation, gender, and career stage, with each group including both subject-matter specialists and participants from outside the focal theme. Themes C, D, and E, holding 65% of all questions, were each analysed by a dedicated group. The other six themes were paired (A+B; F+G; H+I) and allocated to the three remaining discussion groups to balance workload (See Supplementary Data).

Within each group, participants reviewed all questions assigned to their theme(s) and collaboratively identified a shortlist of up to five priority questions. Shortlisting was guided by consideration of scientific importance, novelty, feasibility, and relevance to conservation, restoration, and policy outcomes. Experts were asked to prioritise questions that could most effectively advance blue carbon science, drive change and translate into measurable outcomes for conservation, restoration and policy. To guide this, they were prompted to imagine having ample resources (e.g., US$100 million), transdisciplinary expertise, and a decade to implement each question at scale, encouraging them to focus on questions with high potential for practical impact rather than descriptive research.

Supplementary Text S3. Ranking and refinement of priority questions

Following the independent and anonymous ranking of the 25 questions (Table S2), a structured plenary discussion was held to refine wording and clarity of the top 10 ranked questions, without altering rank order. The lead author (P.I. Macreadie) facilitated the session, presenting the questions in rank order, beginning with the highest scores. Each question was read aloud to confirm comprehension, with theme leaders invited to provide clarification or context as needed. This ensured all participants shared a consistent understanding before the discussion commenced. Open discussion then drew on the diversity of expertise and perspectives in the room to interrogate assumptions and improve wording. The ranking order from the *Mentimeter* scoring exercise was retained throughout; no re-prioritisation occurred. However, some overlapping elements from lower-ranked questions were incorporated into related higher-ranked ones to improve coherence and breadth.

Supplementary Table S1. Comparison of the top research questions in blue carbon science identified in *The Future of Blue Carbon Science*[11] (Macreadie et al. 2019) and the present study, organised into nine thematic areas. Each question is assigned to one thematic category, with colour-coding used for clarity. **(A) Boundaries & Definitions**: Clarifying scope and terminology of blue carbon; **(B) Emerging BCEs**: Including non-classical, emerging blue carbon ecosystems; **(C) Prediction**: Modelling variability and climate change, forecasting, scaling, and drivers; **(D) Measurement**: Inventories, mapping, monitoring, and data management; **(E) Crediting & Standards**: Carbon credit rules and MRV requirements; **(F) Co-Benefits**: Non-carbon services like biodiversity, coastal protection, and nutrient cycling; **(G) Communication**: Conduct, awareness, transparent messaging, managing expectations; **(H) Finance & Markets**: Investment, project affordability, feasibility, and financing mechanism; **(I) Social & Policy**: Communities, equity, policy frameworks, global agendas, and governance.

Q	Macreadie et al. 2019	This study
Q1	How does climate change impact carbon accumulation in mature blue carbon ecosystems and during their restoration? **C. Prediction**	How can we manage blue carbon ecosystems while supporting the livelihoods of coastal communities? **I. Social & Policy**
Q2	How does disturbance affect the burial fate of blue carbon? **C. Prediction**	How can we develop affordable, high-quality methods for implementing restoration? **H. Finance & Markets**
Q3	What is the effect of different restoration approaches on carbon stocks and sequestration rates in blue carbon ecosystems? **C. Prediction**	Can we forecast the future GHG balance of blue carbon ecosystems in response to global change? **C. Prediction**
Q4	How do changes in sediment supply affect carbon sequestration in blue carbon ecosystems? **C. Prediction**	How can we improve estimates of human pressures and management on carbon cycling of blue carbon ecosystems? **C. Prediction**
Q5	How do nutrient inputs affect the sequestration capacity of blue carbon ecosystems? **C. Prediction**	How can we advance natural capital accounting in blue carbon ecosystems to include a more comprehensive range of co-benefits and trade-offs? **F. Co-benefits**
Q6	How can organic matter sources be estimated in blue carbon sediments? **C. Prediction**	Which innovative techniques, analytical tools and new data or proxies may improve the accuracy of blue carbon flux estimates? **D. Measurement**
Q7	What are the roles of carbonate production and dissolution in blue carbon ecosystems in the context of carbon sequestration? **C. Prediction**	Can we simplify blue carbon crediting, while maintaining appropriate integrity standards? **E. Crediting & Standards**
Q8	How can macroalgal contributions to carbon sequestration be quantified and integrated into blue carbon budgets? **B. Emerging BCEs**	Which regions and flux types need priority measurement to improve blue carbon budgets? **D. Measurement**
Q9	What are the emissions implications of disturbed blue carbon ecosystems, particularly regarding methane and nitrous oxide? **C. Prediction**	How can we enhance the accuracy of upscaling BC estimates across scales? **C. Prediction**
Q10	How can improved mapping and monitoring techniques enhance blue carbon science and policy applications? **D. Measurement**	How can we ensure blue carbon data and communication methods effectively inform climate policy? **I. Social & Policy**

Supplementary Table S2. Ranking scores of the blue carbon (BC) questions shortlisted by 30 global blue carbon experts. A total of 116 questions were submitted, grouped into nine thematic categories, and refined through discussion to these 25 questions. Participants then ranked these questions anonymously via the *Mentimeter* software platform, with scores ranging from 1 (lowest priority) to 100 (highest priority). For each question, the score represents the mean priority value assigned by the experts, with the top 10 ranked questions being presented and discussed in the manuscript. The exact wording of some of the top 10 questions was slightly edited during the review process, so both the revised and original (displayed in brackets) are provided. Each question is assigned to one of the nine thematic categories, with colour-coding for clarity. **(A) Boundaries & Definitions**: Clarifying scope and terminology of blue carbon; **(B) Emerging BCEs**: Including non-classical, emerging blue carbon ecosystems; **(C) Prediction**: Modelling variability and climate change, forecasting, scaling, and drivers; **(D) Measurement**: Inventories, mapping, monitoring, and data management; **(E) Crediting & Standards**: Carbon credit rules and MRV requirements; **(F) Co-Benefits**: Non-carbon services like biodiversity, coastal protection, and nutrient cycling; **(G) Communication**: Conduct, awareness, transparent messaging, managing expectations; **(H) Finance & Markets**: Investment, project affordability, feasibility, and financing mechanism; **(I) Social & Policy**: Communities, equity, policy frameworks, global agendas, and governance.

Q	**Revised Question** [Original wording]	**Mean Score**
1	**How can we manage blue carbon ecosystems while supporting the livelihoods of coastal communities?** [How can we effectively manage and conserve BCE while ensuring the prosperity of coastal communities reliant on these ecosystems for their livelihoods?]<br>**I. Social & Policy**	84
2	**How can we develop affordable, high-quality methods for implementing restoration?** [How can we develop economically affordable, yet high-quality restoration methodologies tailored for developing countries?]<br>**H. Finance & Markets**	84
3	**Can we forecast the future GHG balance of blue carbon ecosystems in response to global change?** [Do we have the data and mechanistic understanding to forecast the future GHG balance of BCE in response to change?]<br>**C. Prediction**	83
4	**How can we improve estimates of human pressures and management on carbon cycling of blue carbon ecosystems?** [What are the scientific/technical advances needed to quantify the effects of human-induced land use, land-use change, and forestry (LULUCF) and restoration on BC cycling?]<br>**C. Prediction**	81
5	**How can we advance natural capital accounting in blue carbon ecosystems to include a more comprehensive range of co-benefits and trade-offs?** [How do we ensure that natural capital accounting in BCEs includes their full range of co-benefits and trade-offs?]<br>**F. Co-benefits**	81
6	**Which innovative techniques, analytical tools and new data or proxies may improve the accuracy of blue carbon flux estimates?** [What innovative techniques, analytical tools and new data or proxies are needed to accelerate the accuracy of BC flux and between/beyond BC ecosystems estimation?]<br>**D. Measurement**	78
7	**Can we simplify blue carbon crediting, while maintaining appropriate integrity standards?**<br>**E. Crediting & Standards**	77
8	**Which regions and flux types need priority measurement to improve blue carbon budgets?** [Are we measuring the right things to ensure a conservative estimate for carbon uptake and long-term removal?]<br>**D. Measurement**	74

9	**How can we enhance the accuracy of upscaling blue carbon estimates across scales?** [Can we improve the accuracy of BCE change when upscaling by identifying key BC quantification processes that are scale independent?] **C. Prediction**	71
10	**How can we ensure blue carbon data and communication methods effectively inform climate policy?** [Are current BC data collection and communication approaches effectively articulated with the needs of climate mitigation policy?] **I. Social & Policy**	71
11	What specific policy measures are necessary for integrating BC (and emerging) ecosystems into climate mitigation initiatives effectively? **I. Social & Policy**	70
12	How to incorporate and reduce uncertainty in BC project reporting for implementation by determining minimum data quantity, frequency and type? **E. Crediting & Standards**	70
13	What is the minimum amount of sampling needed for scientifically robust scaled up BC estimates? **D. Measurement**	68
14	Scaling: What are the key processes to quantify BC features that are unique to the scale of the application? Which are scale independent? **C. Prediction**	67
15	How do we maximize available data and increase partnerships for data transparency and trust? **E. Crediting & Standards**	67
16	Is a cross-system BC framework a necessary lens? **A. Boundaries & Definitions**	67
17	What are research priorities and policy developments needed for Emerging BCEs? **B. Emerging BCEs**	67
18	Can we develop innovative techniques to measure and estimate 4D carbon fluxes between and beyond BC ecosystems? **D. Measurement**	66
19	Seaweed BC: why have we not progressed further and what science do we need? **B. Emerging BCEs**	66
20	Can we streamline the BC estimation processes for scientifically robust BC estimates? **D. Measurement**	65
21	Should life-cycle analysis be incorporated into all BC projects? **B. Emerging BCEs**	60
22	How do we improve awareness, accountability and communication of BC actions? **G. Communication**	60
23	Are financial incentives and benefits sufficient to attract, engage, and sustain broad community involvement in conservation efforts? **I. Finance & Markets**	60
24	How to implement the IUCN standards for nature-based solutions in new BC ecosystem projects? **E. Crediting & Standards**	56
25	What are the social, cultural, and economic impacts resulting from the retreat of mangroves due to sea level rise? **I. Social & Policy**	54
