# Supplementary material for: Priority questions for the next decade of blue carbon science
Source: Nat Ecol Evol. 2026 Mar 24;10(4):751–64. doi: 10.1038/s41559-026-03020-6 (PMC13076200; doi:10.1038/s41559-026-03020-6)
Supplement: Supplementary file 2 — Reporting Summary [file 41559_2026_3020_MOESM2_ESM.pdf]

nature portfolio

Corresponding author(s): Peter I. Macreadie

Last updated by author(s): Feb 5, 2026

Reporting Summary

Nature Portfolio wishes to improve the reproducibility of the work that we publish. This form provides structure for consistency and transparency in reporting. For further information on Nature Portfolio policies, see our Editorial Policies and the Editorial Policy Checklist.

Statistics

For all statistical analyses, confirm that the following items are present in the figure legend, table legend, main text, or Methods section.

n/a	Confirmed	
☐	☒	The exact sample size (*n*) for each experimental group/condition, given as a discrete number and unit of measurement
☐	☒	A statement on whether measurements were taken from distinct samples or whether the same sample was measured repeatedly
☒	☐	The statistical test(s) used AND whether they are one- or two-sided<br>*Only common tests should be described solely by name; describe more complex techniques in the Methods section.*
☒	☐	A description of all covariates tested
☒	☐	A description of any assumptions or corrections, such as tests of normality and adjustment for multiple comparisons
☐	☒	A full description of the statistical parameters including central tendency (e.g. means) or other basic estimates (e.g. regression coefficient) AND variation (e.g. standard deviation) or associated estimates of uncertainty (e.g. confidence intervals)
☒	☐	For null hypothesis testing, the test statistic (e.g. *F*, *t*, *r*) with confidence intervals, effect sizes, degrees of freedom and *P* value noted<br>*Give P values as exact values whenever suitable.*
☒	☐	For Bayesian analysis, information on the choice of priors and Markov chain Monte Carlo settings
☒	☐	For hierarchical and complex designs, identification of the appropriate level for tests and full reporting of outcomes
☒	☐	Estimates of effect sizes (e.g. Cohen's *d*, Pearson's *r*), indicating how they were calculated

Our web collection on statistics for biologists contains articles on many of the points above.

Software and code

Policy information about availability of computer code

Data collection	Data were collected using Mentimeter, Google Sheets and Microsoft Excel.
Data analysis	Ranking was done within Mentimeter. Modes were calculated in Microsoft Excel. Map was prepared in ArcGIS.

For manuscripts utilizing custom algorithms or software that are central to the research but not yet described in published literature, software must be made available to editors and reviewers. We strongly encourage code deposition in a community repository (e.g. GitHub). See the Nature Portfolio guidelines for submitting code & software for further information.

Data

Policy information about availability of data

All manuscripts must include a data availability statement. This statement should provide the following information, where applicable:

- Accession codes, unique identifiers, or web links for publicly available datasets
- A description of any restrictions on data availability
- For clinical datasets or third party data, please ensure that the statement adheres to our policy

All data generated for this study are available in the file Supplementary Data 1.xlsx

Research involving human participants, their data, or biological material

Policy information about studies with human participants or human data. See also policy information about sex, gender (identity/presentation), and sexual orientation and race, ethnicity and racism.

Reporting on sex and gender	na
Reporting on race, ethnicity, or other socially relevant groupings	na
Population characteristics	na
Recruitment	na
Ethics oversight	na

Note that full information on the approval of the study protocol must also be provided in the manuscript.

Field-specific reporting

Please select the one below that is the best fit for your research. If you are not sure, read the appropriate sections before making your selection.

☐ Life sciences ☐ Behavioural & social sciences ☒ Ecological, evolutionary & environmental sciences

For a reference copy of the document with all sections, see nature.com/documents/nr-reporting-summary-flat.pdf

Ecological, evolutionary & environmental sciences study design

All studies must disclose on these points even when the disclosure is negative.

Study description	Horizon scan exercise involving an interdisciplinary team of 30 global experts in blue carbon science and policy
Research sample	30 blue carbon experts from 20 countries, representing academic, governmental, and non-governmental institutions.
Sampling strategy	sample size = 30 scientists across diverse expertise, geography, gender balance, and career stages.
Data collection	The Research priorities were gathered through an interactive voting system (Mentimeter), followed by group discussions and anonymous ranking. An additional online ranking exercise was conducted through Google Sheets to asses each of the top 10 questions according to four variables: time scale, cost, research complexity, and policy relevance.
Timing and spatial scale	The initial workshop took place from 13–16 November 2023 at the IAEA headquarters in Vienna, Austria. The additional ranking exercise took place from 20 - 30 November 2024 online in Microsoft Excel.
Data exclusions	Duplicate submissions were consolidated before thematic categorization and ranking.
Reproducibility	The methodology follows established priority-setting frameworks by Sutherland et al 2011(Methods for collaboratively identifying research priorities and emerging issues in science and policy) and Seddon et al. 2014 (Looking forward through the past: identification of 50 priority research questions in palaeoecology. Journal of Ecology 102, 256-267); and can be replicated in similar expert-driven exercises.
Randomization	Participants were assigned to discussion groups ensuring diverse representation across expertise, geography, and demographic background.
Blinding	Poll results were hidden during voting to minimize bias, and rankings were conducted independently and anonymously.

Did the study involve field work? ☐ Yes ☒ No

Reporting for specific materials, systems and methods

We require information from authors about some types of materials, experimental systems and methods used in many studies. Here, indicate whether each material, system or method listed is relevant to your study. If you are not sure if a list item applies to your research, read the appropriate section before selecting a response.

Materials & experimental systems

n/a	Involved in the study
☒	☐ Antibodies
☒	☐ Eukaryotic cell lines
☒	☐ Palaeontology and archaeology
☒	☐ Animals and other organisms
☒	☐ Clinical data
☒	☐ Dual use research of concern
☒	☐ Plants

Methods

n/a	Involved in the study
☒	☐ ChIP-seq
☒	☐ Flow cytometry
☒	☐ MRI-based neuroimaging

Plants

Seed stocks

Report on the source of all seed stocks or other plant material used. If applicable, state the seed stock centre and catalogue number. If plant specimens were collected from the field, describe the collection location, date and sampling procedures.

Novel plant genotypes

Describe the methods by which all novel plant genotypes were produced. This includes those generated by transgenic approaches, gene editing, chemical/radiation-based mutagenesis and hybridization. For transgenic lines, describe the transformation method, the number of independent lines analyzed and the generation upon which experiments were performed. For gene-edited lines, describe the editor used, the endogenous sequence targeted for editing, the targeting guide RNA sequence (if applicable) and how the editor was applied.

Authentication

Describe any authentication procedures for each seed stock used or novel genotype generated. Describe any experiments used to assess the effect of a mutation and, where applicable, how potential secondary effects (e.g. second site T-DNA insertions, mosiacism, off-target gene editing) were examined.
